# Nanoscale elemental and morphological imaging of nitrogen-fixing cyanobacteria

**DOI:** 10.1093/mtomcs/mfae040

**Published:** 2024-09-13

**Authors:** Bobby G Duersch, Steven A Soini, Yanqi Luo, Xiaoyang Liu, Si Chen, Vivian M Merk

**Affiliations:** Department of Chemistry and Biochemistry, Department of Ocean and Mechanical Engineering, Florida Atlantic University, Boca Raton, FL, USA; Department of Chemistry and Biochemistry, Department of Ocean and Mechanical Engineering, Florida Atlantic University, Boca Raton, FL, USA; Advanced Photon Source, Argonne National Laboratory, Lemont, IL, USA; Advanced Photon Source, Argonne National Laboratory, Lemont, IL, USA; Advanced Photon Source, Argonne National Laboratory, Lemont, IL, USA; Department of Chemistry and Biochemistry, Department of Ocean and Mechanical Engineering, Florida Atlantic University, Boca Raton, FL, USA

**Keywords:** elemental mapping, cyanobacteria, synchrotron X-ray fluorescence, energy-dispersive X-ray analysis

## Abstract

Nitrogen-fixing cyanobacteria bind atmospheric nitrogen and carbon dioxide using sunlight. This experimental study focused on a laboratory-based model system, *Anabaena* sp., in nitrogen-depleted culture. When combined nitrogen is scarce, the filamentous prokaryotes reconcile photosynthesis and nitrogen fixation by cellular differentiation into heterocysts. To better understand the influence of micronutrients on cellular function, 2D and 3D synchrotron X-ray fluorescence mappings were acquired from whole biological cells in their frozen-hydrated state at the Bionanoprobe, Advanced Photon Source. To study elemental homeostasis within these chain-like organisms, biologically relevant elements were mapped using X-ray fluorescence spectroscopy and energy-dispersive X-ray microanalysis. Higher levels of cytosolic K^+^, Ca^2+^, and Fe^2+^ were measured in the heterocyst than in adjacent vegetative cells, supporting the notion of elevated micronutrient demand. P-rich clusters, identified as polyphosphate bodies involved in nutrient storage, metal detoxification, and osmotic regulation, were consistently co-localized with K^+^ and occasionally sequestered Mg^2+^, Ca^2+^, Fe^2+^, and Mn^2+^ ions. Machine-learning-based k-mean clustering revealed that P/K clusters were associated with either Fe or Ca, with Fe and Ca clusters also occurring individually. In accordance with XRF nanotomography, distinct P/K-containing clusters close to the cellular envelope were surrounded by larger Ca-rich clusters. The transition metal Fe, which is a part of nitrogenase enzyme, was detected as irregularly shaped clusters. The elemental composition and cellular morphology of diazotrophic *Anabaena* sp. was visualized by multimodal imaging using atomic force microscopy, scanning electron microscopy, and fluorescence microscopy. This paper discusses the first experimental results obtained with a combined in-line optical and X-ray fluorescence microscope at the Bionanoprobe.

## Graphical Abstract

**Graphical Abstract:**
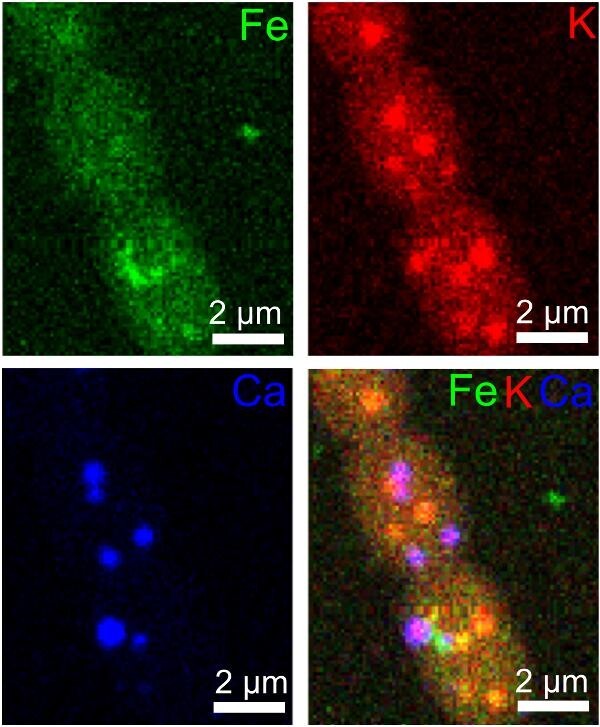
2D Synchrotron X-ray fluorescence mapping visualizing Fe, K and Ca distribution in frozen-hydrated nitrogen-fixing cyanobacteria.

## Introduction

Cyanobacteria are ancient photosynthetic prokaryotes that evolved in terrestrial and aquatic ecosystems over 2.4 billion years ago.^1^ The cyanobacteria *Anabaena* sp. were among the first organisms on earth to have developed multicellularity by turning vegetative cells into heterocysts, presenting an early example of cellular differentiation in evolution.^2^ To the present day, nitrogen-fixing cyanobacteria release molecular nitrogen into aquatic ecosystems, thereby contributing to the eutrophication of natural waters and paving the way for the excessive growth of other toxic, bloom-forming cyanobacteria, such as *Microcystis aeruginosa*.^3^ Apart from their problematic impact on the phytoplankton population, *Anabaena* sp. produces neurotoxins harmful to humans, local wildlife, and livestock.^4^

**Fig. 1:**
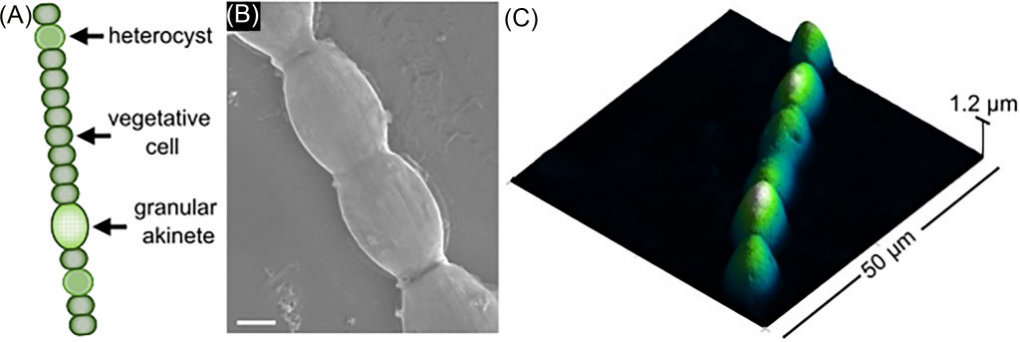
Cellular morphology of filamentous nitrogen-fixing cyanobacteria. (A) Schematic of *Anabaena* sp. containing beadlike vegetative cells, roundish heterocysts, and granular akinetes. (B) Field-emission Scanning Electron Micrograph. Scale bar equals 2 μm. (C) Topographic Atomic Force Micrograph of *Anabaena* sp. filament.

When combined nitrogen is scarce, diazotrophic cyanobacteria reconcile two biochemically incompatible processes, photosynthesis and nitrogen fixation. To this end, cyanobacteria have developed different mechanisms to avoid the oxidation of the oxygen-labile nitrogenase core: In the absence of fixed nitrogen, unicellular strains, such as *Synechococcus elongatus*,^5^,^6^ perform oxygen-evolving photosynthesis at daylight and nitrogen fixation at night, while filamentous strains, such as *Anabaena* sp.,^7^ separate photosynthesis and nitrogen fixation in space by using morphologically and metabolically differentiated heterocyst cells that contain very low O_2_ concentrations (Fig. 1A).^8^ This micro-oxic environment is thought to be created by increased respiration, inactivation of the O_2_-producing photosystem II, and formation of a thickened envelope outside of the heterocyst cell wall.^9^,^10^ While all cells in the filament are interconnected by a continuous periplasmic space, designated communicating channels named microplasmodesmata penetrate the septum between adjacent cells, enabling nitrogen, nutrient, and metabolite transport between heterocysts and vegetative cells.^9^,^10^ The reduction of nitrogen to ammonia is a highly energy-demanding reaction, requiring 16 ATP units per nitrogen molecule.^11^ Carbohydrates synthesized in vegetative cells are transported to heterocysts, while fixed nitrogen produced by the heterocysts is moved to vegetative cells. Apart from macronutrients, cyanobacteria have an increased demand for micronutrients. As markers of enzymatic activity or metalloproteins, trace metals reflect biological redox process and regulate the expression of genes involved in biological processes, such as photosynthesis or N_2_-fixation.^12–14^ Trace metals establish the active sites of metallocofactors and metalloproteins, such as nitrogenase (Fe, Mo), ferric uptake regulator protein (Fe) or chlorophyll (Mg).^12–14^ For instance, photosynthetic cyanobacteria such as *Anabaena* sp., require at least 10 times more Fe than non-photosynthetic bacteria like *Escherichia coli* in their growth medium.^15^ Nitrogen deprivation in the growth medium is known to increase the demand of Fe^15^,^16^ and Ca.^2^,^17–20^ While needed in much lower concentrations, Mo plays a crucial role in nitrogen fixation as part of the active center of nitrogenase.^14^,^21–25^ Yet, despite the large body of literature on cyanobacteria, there are fundamental gaps in our knowledge about their elemental uptake, metal homeostasis, and intercellular transport. Previous research indicated that diazotrophic cyanobacteria reduce atmospheric dinitrogen by increasing gene expression under nitrogen-free growth conditions.^16^ However, it is yet poorly understood how the cellular machinery responds to fixed nitrogen deprivation.

In the present paper, we applied state-of-the-art synchrotron X-ray fluorescence imaging (XRF) and cryopreservation tools to investigate the ionome, the cellular trace element composition of nitrogen-fixing cyanobacteria grown under nitrogen starvation. This experimental study focused on an axenic laboratory-based model system, the filamentous freshwater cyanobacterium *Anabaena* sp.,^5^ which relies on cellular differentiation to separate photosynthesis from nitrogen fixation.^26^ Previous research indicated that micronutrients, such as Fe, Mo, Cu, Co, Ni, Mn, and K, are key to the growth and cellular function of cyanobacteria.^7^,^27–30^ Environmental factors, such as micronutrient limitation or supplementation, are known to influence nitrogen-metabolic pathways in filamentous nitrogen-fixing cyanobacteria, specifically cellular differentiation.^31^,^32^ While previous studies quantified the cellular uptake of trace elements at the bulk level,^33^,^34^ detailed information on the subcellular elemental distribution is still lacking. Using energy-dispersive X-ray microanalysis in the electron microscope, high levels of Mg, Si, P, S, Cl, K, and Ca were detected in the cyanobacteria *Anabena flos-aquae* collected from a eutrophic freshwater lake, but the subcellular element localization was not specified.^34^

The Bionanoprobe, currently beamline 9-ID-B/C at the Advanced Photon Source, Argonne National Laboratory, is dedicated to hard X-ray fluorescence microscopy of intact, frozen-hydrated biological samples with sub-100 nm spatial resolution and high chemical sensitivity toward multiple trace elements.^35^ Due to the small size of these simple multicellular organisms, nanoscale resolution is essential to visualize the subcellular ultrastructure. Cryogenic storage, sample transfer, and measurements at a temperature close to the temperature of liquid nitrogen are critical for preserving the integrity and elemental composition of sensitive biological cells.^35^ Apart from synchrotron-based XRF imaging, morphological differentiation, and cell division of *Anabaena* sp. after combined nitrogen starvation were studied using Atomic Force Microscopy, Scanning, and Transmission Electron Microscopy. Furthermore, this paper discusses the first experimental results acquired with a combined in-line optical and X-ray fluorescence microscopic set-up implemented at the Bionanoprobe.

## Materials and methods

### *Anabaena sp.* cultures

Axenic *Anabaena* sp. cultures (UTEX #2576, aka *Nostoc* sp. PCC 7120) were purchased from the Culture Collection of Algae at The University of Texas at Austin (UTEX) and maintained in a nitrate-free freshwater growth medium BG-11(-N) medium in sterile 150 ml Erlenmeyer flasks. The cultures were kept at 20°C under constant agitation in a Faithful Instrument cooling shaking incubator.

### Scanning electron microscopy preparation and imaging

*Anabaena* sp. cells were washed three times with *tris*-HCl buffer and centrifuged between each washing. Cells were fixed in 2% glutaraldehyde at 4°C for 4 h and resuspended before pipetting onto a silica wafer, then mounted onto an Al specimen holder and left to air dry. Prior to scanning electron microscopy (SEM) analysis, samples were sputter-coated with a 10–15 nm thin layer of Pt under an argon atmosphere at 8 mA for 30 s using a MicroNanoTools MNT-JS1600 plasma sputtering coater. Samples were imaged at an acceleration voltage of 15 kV using a JEOL FS100 field-emission scanning electron microscope (Fig. 1B).

### Atomic force microscopy

Fresh *Anabaena* sp. cells suspended in culture medium were dropped on  <100>  oriented Si wafer chips (Ted Pella). Samples were characterized using an atomic force microscopy (AFM) Workshop TT-2 atomic force microscope with 50 µm scanner (Fig. 1C). Samples were scanned in tapping mode using AFM Workshop ACLA-10-W probes with a rectangular Sb-doped Si cantilever with reflective Al coating and a 58 N/m spring constant. Topography and phase images were post-processed using the Gwyddion (v. 2.59) software package. The standard processing procedure included mean plane subtraction to level data and removal of polynomial background.

### Confocal fluorescence microscopy

**Fig. 2:**
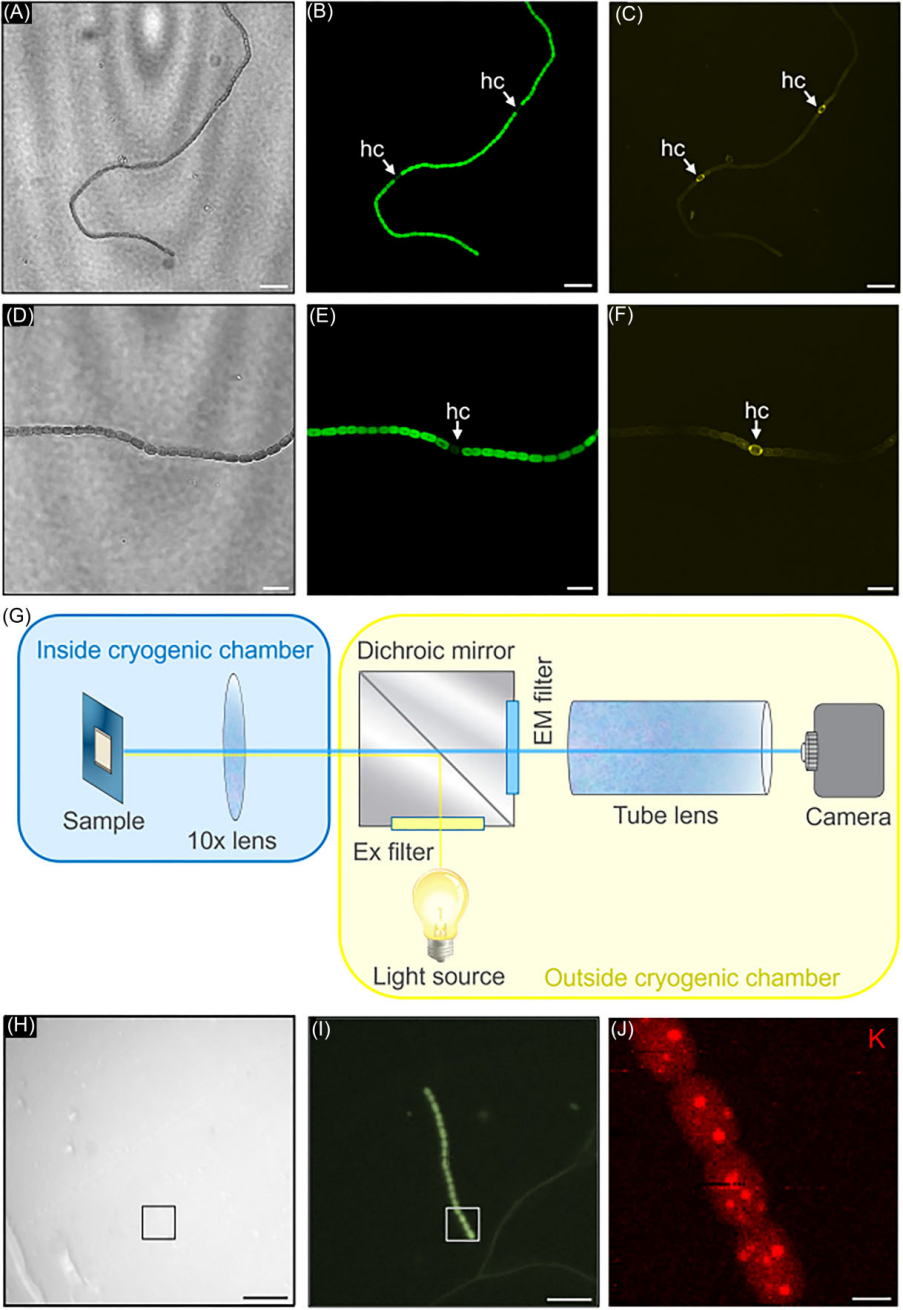
*Ex situ* optical confocal fluorescence microscopy of a differentiated *Anabaena* sp. filament. (A and D) Bright-field images. (B and E) Chlorophyll autofluorescence. (C and F) Heterocysts marked with BODIPY™ fluorescent dye. Scalebars in (A–F) correspond to 10 μm. (G) Correlative optical fluorescence and X-ray fluorescence setup at Bionanoprobe beamline. (H) In-line bright-field light micrograph, (I) in-line chlorophyll autofluorescence and (J) synchrotron X-ray fluorescence mapping of potassium distribution in vegetative *Anabaena* sp. cells. The K concentration color bar is scaled as 0–20.5 μg cm^-2^. Scale bars in (H and I) correspond to 20 μm and in (J) to 2 μm.

Heterocysts were stained with the Invitrogen BODIPY™ FL C_12_ dye, 4,4-Difluoro-1,3,5,7-Tetramethyl-4-Bora-3a,4a-Diaza-s-Indacene (Thermo Fisher Scientific), which is characterized by a 488 nm excitation and 510 nm emission line. A volume of 0.5 ml cell culture was mixed with 2.5 μl of a 1 mM BODIPY™ solution in dimethyl sulfoxide (Thermo Fisher Scientific), corresponding to a final DMSO concentration of 70 mmol l^−1^ in the cell culture aliquot used for optical fluorescence imaging. *Ex-situ* confocal fluorescence micrographs (Fig. 2) were acquired with a Nikon A1R confocal system with N-SIM E on an Eclipse Ti2 inverted microscope. Chlorophyll autofluorescence was excited at 488 and 561 nm to give a broad emission  >665 nm.

### Preparation of cells for SR-XRF analysis

Low-stress SiNx windows (Norcada) were used as sample substrates. Live *Anabaena* sp. cells were drop-cast onto SiNx windows and vitrified by plunge freezing in liquid nitrogen. Excess water was blotted off with a Kimwipe prior to plunge freezing in liquid nitrogen. The SiNx windows were kept in customized holders at  −196°C and cryo-shipped to the Advanced Photon Source, Argonne National Laboratory, Lemont, IL, USA.

### SR-XRF 2D and 3D mappings and area concentrations

**Fig. 3:**
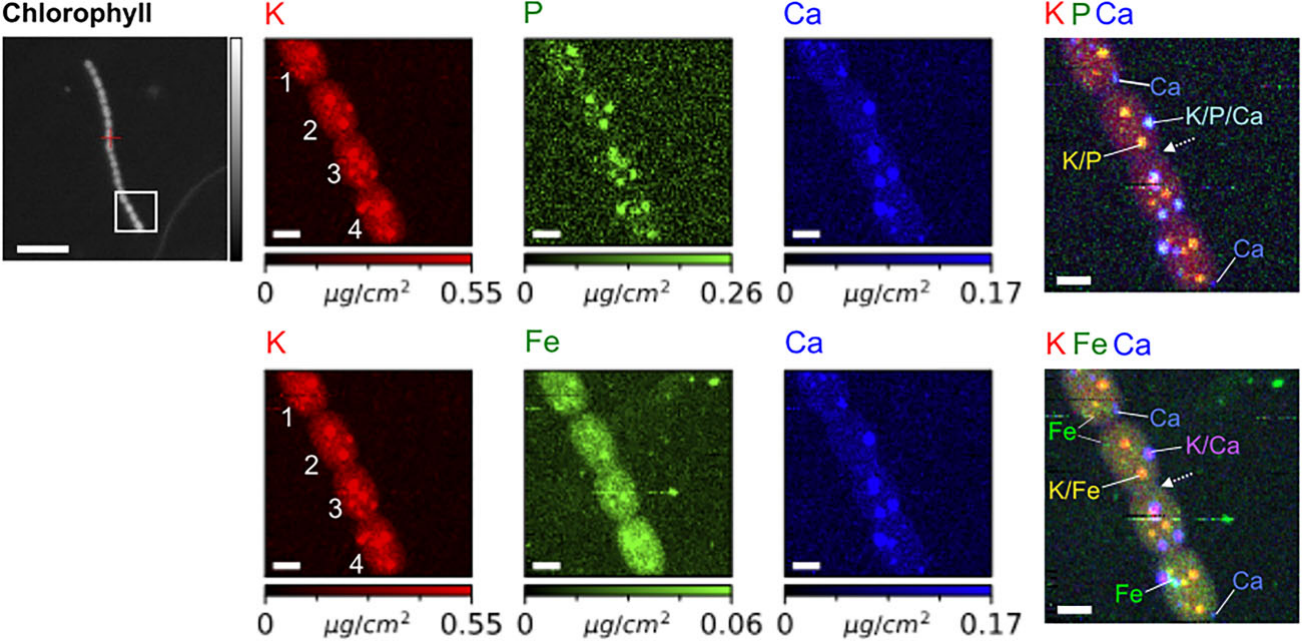
High-resolution X-ray fluorescence mapping of filamentous *Anabaena* sp. containing long chain of vegetative cells. Elemental mappings of K, P, Fe, and Ca are displayed. Three-element overlays reveal distinct K/P/Fe and K/P/Ca clusters and small isolated Ca and Fe clusters. The blurred septal junction between two central cells is marked with a dashed arrow. Color bars were scaled to represent the 98th percentile of the concentration of each element. Scale bars equal 2 μm in XRF panels and 10 μm in the chlorophyll panel.

Scanning X-ray fluorescence mappings were collected in conjunction with differential phase-contrast imaging at the 9-ID-B/C end station (Bionanoprobe)^36^ with an incident photon energy of 10 keV. Detected elements include Z = 13 (Al)—30 (Zn), specifically P, S, K, Ca, Cr, Mn, Fe, Co, Ni, Cu, and Zn. Target cells were located using a light microscope with optical fluorescence at the beamline. An inline optical fluorescence microscope at the beamline was equipped with an X-cite NOVEM 9-channel LED light source (475 nm channel), a dichroic mirror as beam splitter and external filters. Coarse scans of 500 nm step size and 100 ms pixel^−1^ dwell time allowed us to localize filaments on SiNx windows. Intermediate resolution scans with 300 nm step size and 200 ms pixel^−1^ dwell time provided 2D mappings of individual filaments. High-resolution scans were collected from regions of interest with a step size of 100 nm and a dwell time of 200 ms pixel^−1^. Entire X-ray fluorescence spectra were acquired for each pixel using a collimated seven-element Si-drift detector (Vortex-ME7, Hitachi High-Tech Science America, USA). The background was approximated using a peak-stripping algorithm. Spectral peaks were fitted to an exponentially modified Gaussian curve to determine the peak areas in accordance with fixed ratios of the K_α_ and K_β_ peak areas. Elemental mappings and three-color colocalization mappings were generated using the custom-built MAPS-software.^37^,^38^ X-ray fluorescence counts were converted to area concentrations (g cm^−2^) using peak area to concentration ratios determined from an AXO thin-film standard (AXO DRESDEN GmbH, Dresden, Germany). Two-dimensional XRF elemental mappings were displayed in arbitrary colors with varying concentration scales. Color ranges in which the maximum value represents the 98.0 percentile of the data were used to enhance the image contrast in 2D XRF mappings (Figs. 3 and 7).

A full tomographic dataset was obtained from a total of 51 projections with 100 nm step size and 150 ms/pixel dwell time, using an angular range of −82° to 70°. The 3D tomographic reconstruction was done using an SIRT algorithm in the ASTRA toolbox.^39^ The Tomviz software package was used for volumetric visualization of the 3D tomographic datasets.^40^

For regions of interest (ROI) concentration analysis, the cellular outlines were delineated based on K XRF mappings. K-mean cluster segmentation was applied to Ca XRF mappings to separate Ca-rich clusters from the remaining cell.^41^ For cells containing Ca-rich clusters, a fixed-thresholding value was applied to Ca XRF mappings to locate the coordinates of Ca-rich clusters. These coordinates were masked in Ca, K, and Fe XRF mappings to highlight the cytosolic area outside of Ca-rich clusters. The average concentration was calculated for each region after background subtraction. By using two different methods to determine the outline of Ca-rich clusters, we can decrease the concentration errors from the Ca-rich boundary of the clusters.

The area and correlation coefficients of elemental clusters were calculating from segmented 2D XRF data by identifying isolated ROIs using the open-source Python-based machine learning package Scikit-learn (sklearn),^41^ skimage,^42^ and scipy.^43^ For cluster segmentation, we applied a narrower color range with the maximum value representing the 99.9^th^ percentile of the data, which emphasizes clusters while excluding outliers. Prior to ROI determination, all elemental XRF mappings were marked using the potassium (K) elemental distribution to define the boundary contour of the cytoplasm and to eliminate the contribution from scanned areas outside the bacteria. The unsupervised k-mean clustering method (package in sklearn), applied to individual elemental XRF mappings, was used to estimate the intensity value that separates cluster and non-cluster regions. Once the ROIs were identified, Pearson's R-values (package in SciPy) were computed as the estimated correlation coefficient. The cluster area was calculated by multiplying the total number of pixels classified as clusters by the pixel size, here 0.01 µm^2^.

### Transmission electron microscopy

Samples were dehydrated using an ethanol series (EtOH/H_2_O: 25/75, 50/50, 75/25, 90/10, 100/0, 100/0) and embedded in araldite resin (Embed 812, Electron Microscopy Sciences). Slices of 200 nm were prepared using a Leica Ultracut EM UC7RT ultramicrotome with a 35° DiATOME® ultra diamond knife and placed on formvar-coated 200 mesh Cu grids (PELCO®). Samples were fixed with 1% glutaraldehyde and embedded in an epoxy (Embed 812) resin. A 120 kV JEOL JEM-1400 transmission electron microscope (TEM) equipped with a scanning TEM unit for bright-field and dark-field imaging, and Oxford AZtec energy-dispersive X-ray detector was utilized to study the nanoscale elemental distribution across thin sections.

## Results and discussion

The present study focused on mapping the cellular morphology and quantitative distribution of trace metals involved in photosynthesis, nitrogen metabolism, stress signaling, and nutrient storage in diazotrophic cyanobacteria at the cellular and organelle level. This data provide cues to unravel the cellular and molecular pathways that enable simultaneous photosynthesis and nitrogen fixation in these multicellular organisms. Toward this goal, we quantified and imaged multiple trace elements, such as Fe, K, and Ca, with nanometer resolution.

### *Ex situ* fluorescence and inline optical and X-ray fluorescence imaging

Using *ex situ* confocal fluorescence microscopy, we distinguished between vegetative and heterocyst cells based on their cellular morphology and fluorescence signal. When cells are deprived of combined nitrogen, *Anabaena* sp. produce filamentous chains of tens to hundreds of cells with heterocysts intercalated among vegetative cells in a semiregular fashion.^10^ In the *ex situ* experiment, heterocyst cells were labeled with the fluorescent dye BODIPY™, which does not stain surrounding vegetative cells. As shown in Figs. 1 and 2A–F, vegetative cells are characterized by a truncated shape and intense chlorophyll autofluorescence, while heterocysts appear roundish and less pigmented. Under diazotrophic growth conditions, filaments typically contain 1–2 heterocysts per filament.

A new in-line optical microscope was introduced at the Bionanoprobe to correlate X-ray fluorescence with optical fluorescence (Fig. 2G). This highly configurable inline optical fluorescence setup featured 9-channel X-cite NOVEM LED light sources and various optical filters. Due to poor contrast in brightfield imaging (Fig. 2H), frozen-hydrated *Anabaena* sp. cells were located based on their intense chlorophyll fluorescence signal from the thylakoids. To avoid sample contamination, vegetative cells and heterocysts were distinguished based on chlorophyll autofluorescence.^44^,^45^ By morphological distinction, we discerned vegetative cells at various stages of the cell cycle, including non-diving cells, recently divided cells, or cells undergoing division (Fig. 2I).

### Mapping and quantifying the elemental distribution with nanometer resolution

Previous research indicated that micronutrients, such as Fe, Mo, Cu, Co, Ni, Mn, and K are key to the growth and function of cyanobacteria.^27–30^ In the present study, synchrotron X-ray fluorescence imaging was used to quantitatively map the trace element distribution in cyanobacteria cells. In contrast to optical microscopy, X-ray imaging does not require the use of fluorescent dyes. The cryogenic capabilities enabled a visualization in the near-native state, thereby minimizing sample preparation artefacts, radiation damage, and sample drift.^46^ The presence of leachable ions, such as K^+^, further proves successful cryopreservation and cellular integrity during the experiment. At an incident photon energy of 10 keV, elements from Z = 13 (Al) to Z = 30 (Zn) were quantified, specifically P, S, K, Ca, Cr, Mn, Fe, Co, Ni, Cu, and Zn. Figure S1 shows a filament section containing 10 vegetative cells. A higher resolution XRF mapping from the same filament (Fig. 3) reveals that the two neighboring cells in the center are not clearly separated, indicating recent cell division along the filament axis. As a result, the septal junction separating the two daughter cells is blurred, as marked by a dashed arow in Fig. 3. Experimental evidence pointed toward the existence of cytoplasmic connections that facilitate molecular exchange between cells.^47^ Such cytoplasmic connections between cells are likely to support the exchange of inorganic micronutrients between heterocysts and vegetative cells in the same filament (Fig. 7).

Among the quantified micronutrients, potassium is a highly abundant cation that regulates the physiological metabolic activity in cyanobacteria.^27^ K^+^ fulfills multiple important functions in bacterial cells, such as maintaining turgor pressure and intracellular pH, enzyme activation, gene expression, and stress response regulation.^48^ Previous research demonstrated that K^+^ is a vital micronutrient in *Anabaena* bacterial cultures, in particular in the absence of combined nitrogen.^49^ Among all the detected elements, K was the most abundant micronutrient present in the bacterial cytosol (Figs. 3 and 7). Remarkably, we did not observe any K clusters in the heterocyst, but 2–4 in each vegetative cell. In addition to the overall high levels of intracellular K, we found distinct K clusters co-localized with P in all vegetative cells, which might serve as ion reservoirs for periods of active growth. K clusters measured roughly 0.99 ± 0.48 μm in diameter. Similarly, our previous research involving the unicellular cyanobacteria *Microcystis aeruginosa* found distinct P/K clusters with a mean diameter of 0.45 ± 0.05 μm.^50^ Given the high energy demand of nitrogen fixation, we speculate that size differences in P/K clusters are related to diverging metabolic rates and pathways in unicellular and multicellular, heterocyst-forming cyanobacteria.^51^ The K-rich protrusion next to cell 4 may be cell debris or an inorganic K-containing crystal, as found in SEM (Fig. S4).

**Fig. 4:**
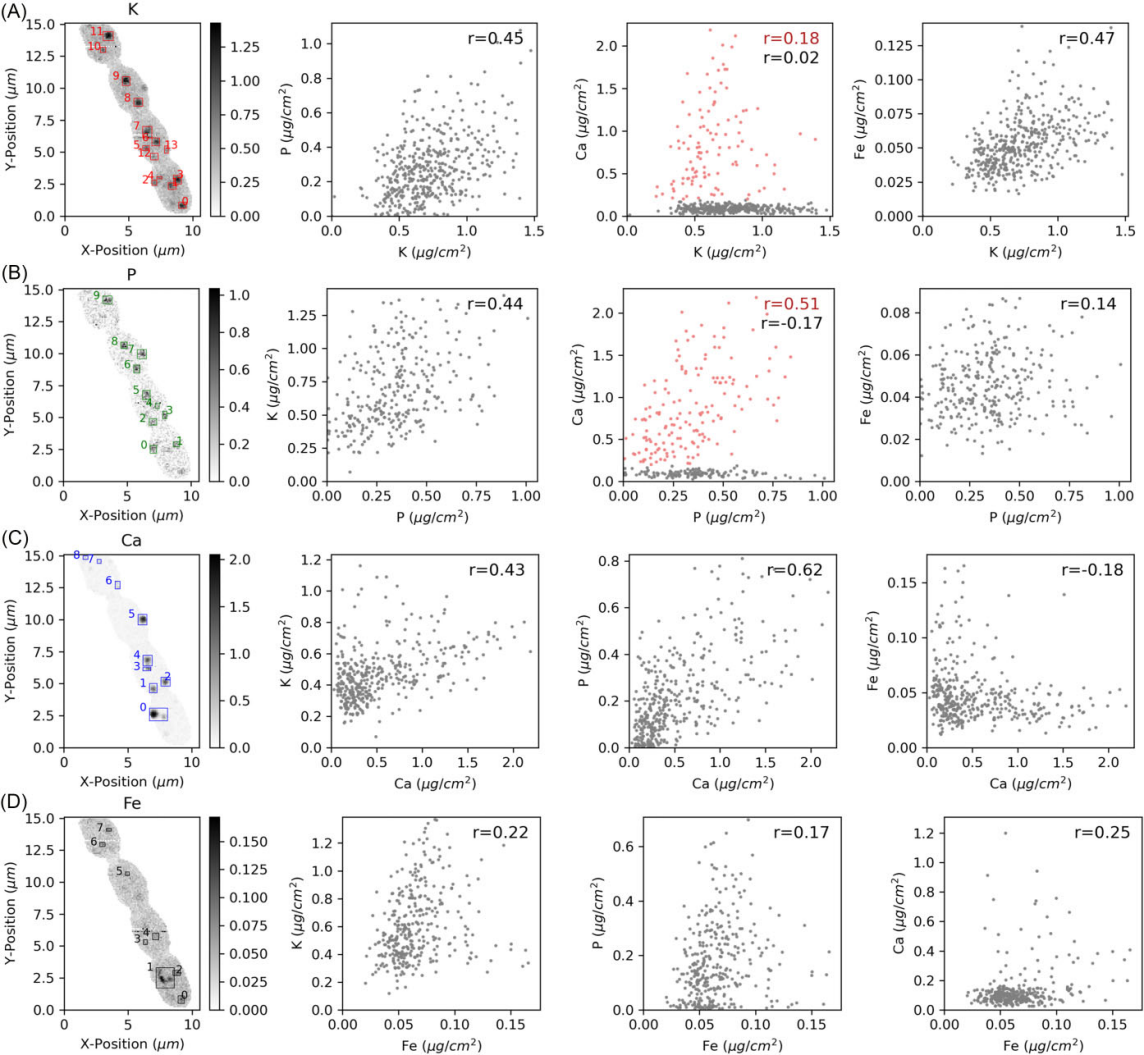
Machine-learning-based image segmentation and cluster analysis of XRF mapping shown in Fig. 3. The bounding boxes detected around individual clusters vary in size and position, depending on the XRF signal of each element. Region of interest (ROI) analysis was based on elemental intensity with Pearson's R value. To emphasize the clusters, color bars were scaled to represent the 99.9th percentile of the concentration of each element.

Phosphorous occurring in the form of inorganic pyrophosphate and polyphosphate, nucleic acids, phospholipids, phosphoproteins, or P-bearing metabolites is involved in a myriad of cellular functions, including catabolic and anabolic metabolic pathways and signaling.^52^ Previous research revealed that *Anabaena* sp. accumulate excess phosphorus in the form of polyphosphate (polyP) inclusions.^53^ Polyphosphate granules are usually stored in acidic calcium-storage vacuoles called acidocalcisomes,^52^,^54^ which serve as an energy source for fueling the cellular metabolism, maintaining the adenylate and metal cation homeostasis, serving as chaperones, modifying protein activity through covalent binding, and contributing to cellular stress regulation.^52^ Due to their anionic nature, polyP is instrumental in binding and sequestering inorganic cations to curb excess intracellular cation concentrations.^52^ It is known that cyanobacteria are capable of concentrating CO_2_ at the site of the carboxylating enzyme ribulose 1,5-bisphosphate carboxylase/oxygenase (RuBisCO) to levels 1000-fold higher than in the ambient environment.^55^ While polyP bodies can occur in other locations of the cyanobacterial cells, they are often found in close proximity to carboxysomes, RuBisCO-encapsulating bacterial microcompartments.^52^ Given the importance of P in cell physiology, P-rich clusters were detected in all imaged vegetative cells (Figs. 3 and 4). Interestingly, two types of P-rich clusters were identified: While K and P were consistently co-localized, Ca was detected only in selected P/K clusters (Fig. 3). Other P/K clusters contained Fe, but no Ca. We applied machine-learning-based image segmentation and unsupervised K-mean clustering to the XRF data displayed in Figs. 3 and 7. The bounding boxes detected around individual clusters varied in size and position, depending on the XRF signal of each element. ROI analysis based on elemental intensity provided the Pearson's R-value (Figs. 4,  S7). We found weak positive correlations (Pearson's R-value ≈ 0.4–0.6) between the elemental concentrations of K-P, K-Ca, K-Fe, and K-P, as visualized in Figure 4. A weak positive correlation was observed in a part of the concentration data from Ca-P clusters, indicating that specific Ca clusters are associated with P, while others are not. Based on the analysis, the elemental concentrations of Ca and Fe were not positively correlated. We further quantified the relative overlap area of two elements per ROI (Figs. S3 and S6). Apart from an intermediate overlap area of the elements K-P, K-Fe, and P-Fe, we found multimodal distributions in the spatial overlap functions, which backs our visual observation that different types of ion-rich clusters exist in *Anabaena* sp. or that some elemental clusters exceed others in size. The varying elemental profiles in the clusters could hint toward different cellular environments that enable nutrient storage, osmotic regulation, and metal detoxification.^33^

As expected, we observe high levels of cytosolic Ca^2+^ in filamentous *Anabaena* sp., as shown in Figs. 3 and 4. Intriguingly, Ca/K/P clusters were not observed within the heterocyst but in vegetative cells, indicating that nutrient storage only happens in vegetative cells. The Ca^2+^ ion employs multiple biological functions, including the perception, communication, and adjustment of cellular responses to environmental changes.^2^,^18^,^32^ For instance, the Ca^2+^-binding protein named Ca^2+^ Sensor EF-hand protein plays an important role in regulating photosynthetic activity in response to changes in carbon and nitrogen availability.^19^ To determine the elemental concentrations within ion-rich clusters as opposed to the surrounding cytosol, the cellular outlines were delineated based on the strong K signal (Fig. S2). Subsequently, K-mean cluster segmentation was applied to Ca XRF mappings to separate Ca-rich clusters from the remaining cell.

**Fig. 5:**
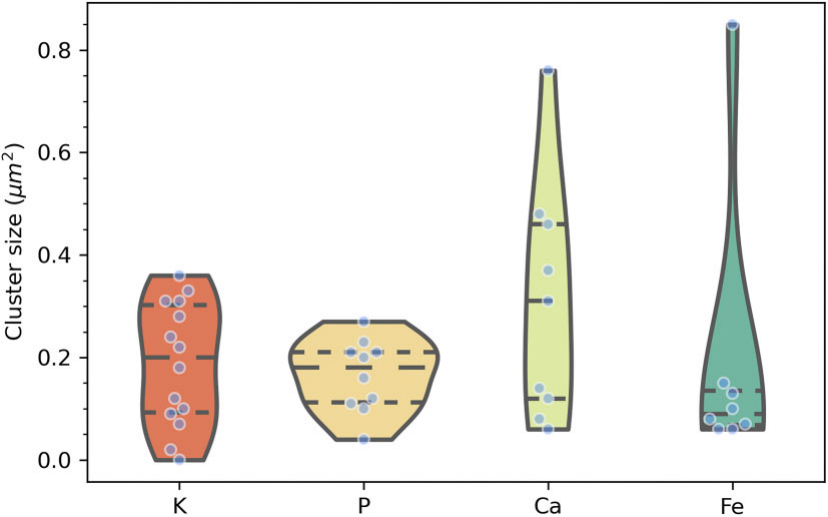
Cluster sizes for K, P, Ca, and Fe elements detected within bounding boxes of Figs. 3 and 4 were plotted as a violin plot. The central dashed line denotes the median. The three dashed lines within each shaded block show the 25th, 50^th^, and 75th percentile of the data from bottom to top.

In the present study, quantitative XRF mappings show slighly higher levels of cytosolic Ca^2+^ in the heterocyst (0.073 μg/cm^2^) than in adjacent vegetative cells (0.029 ± 0.05 μg/cm^2^) outside of ion-rich clusters (Fig. 7), which supports the critical role of Ca^2+^ in heterocyst differentiation, as described in previous research.^2^,^17^,^56^ However, X-ray fluorescence spectroscopy is unsuitable to specify whether cytosolic Ca^2+^ exists in a free or chemically bound form. In addition, we measured higher levels of cytosolic K (0.452 ± 0.18 μg/cm^2^) and Fe (0.095 ± 0.12 μg/cm^2^) in the heterocyst than in vegetative cells outside of ion-rich clusters, where 0.277 ± 0.18 μg/cm^2^ K and 0.027 ± 0.05 μg/cm^2^ Fe were detected. Apart from being present throughout the cellular cytoplasm, Ca is enriched in round globules whose Ca concentrations exceed the cytosolic concentration by several factors. The elemental concentrations varied between vegetative cells, but with 0.029 ± 0.05 μg/cm^2^, the average Ca concentration in the cytosol of vegetative cells 2–5 vastly differed from Ca-rich clusters, which contained 1.337 ± 0.64 μg/cm^2^ (Fig. 4). Our XRF data suggest that Ca^2+^ is associated with P, but its molar concentration and cluster dimension exceed P and K hotspots (Table S1). Using image segmentation, ROIs were delineated around individual clusters depending on the XRF signal of each element (Fig. 4). Cluster sizes were plotted in a violin plot (Figs. 5 and  S8), which displays distributions of numeric data using density curves. We computed mean cluster sizes plus 1.5 x interquartile ranges based on quantitative XRF mappings. It is evident from the violin plot that Ca and Fe clusters come in a broader size range than K and P clusters. However, a Student's *t*-test demonstrated that Ca clusters with a mean size of 0.310 μm^2^ are not significantly larger than K (*P*  = 0.180) or P clusters (*P*  = 0.109) with mean areas of 0.200 and 0.180 μm^2^ at a 95% confidence level. However, these cluster size results contradict previously reported dimensions of acidocalcisomes, which were described as electron-dense spheres with 15–200 nm diameter.^54^ While a broad size dispersion is typical for biological specimens, it is conceivable that elemental hotspots below the pixel size of the XRF mappings, here 100 nm, were not fully resolved. Based on our XRF mappings, nutrient storage within ion-rich clusters primarily occurs within vegetative cells.

In cyanobacteria, polyP granules are thought to serve as inorganic phosphate reservoirs capable of scavenging mono- and divalent cations.^52^ While the chemical environment of Ca^2+^ in the clusters could not be probed with our experimental set-up, their nearly perfect spherical shape may point toward the existence of Ca-rich intravesicular or lipid-bound microcompartments or the deposition of calcium-containing minerals. Blondeau *et al*. observed intracellular amorphous calcium carbonate granules in various cyanobacterial strains.^57^ Even though *Anabaena* sp. was not focus of their study,^57^ it is conceivable that this strain also undergoes intracellular mineralization in the presence of Ca^2+^ levels under standard culture conditions, which tend to be higher than in the environment.

In contrast to vegetative cells, heterocysts are known to have thicker cell walls consisting of a glycolipid layer and a polysaccharide layer, the so-called outer exo-polysaccharide layer (hep layer) and the inner laminated glycolipid layer (hgl layer), which protect the cells from environmental oxygen.^10^,^58^ Comparative chemical analyses demonstrated that the heterocyst wall and mucilage layer of *Anabaena cylindrica* is primarily composed of carbohydrates.^59^ Based on our 2D XRF data (Fig. 7), Ca^2+^ is enriched in the outer heterocyst envelope, probably due to complexation by the outer exo-polysaccharide layer.

**Fig. 6:**
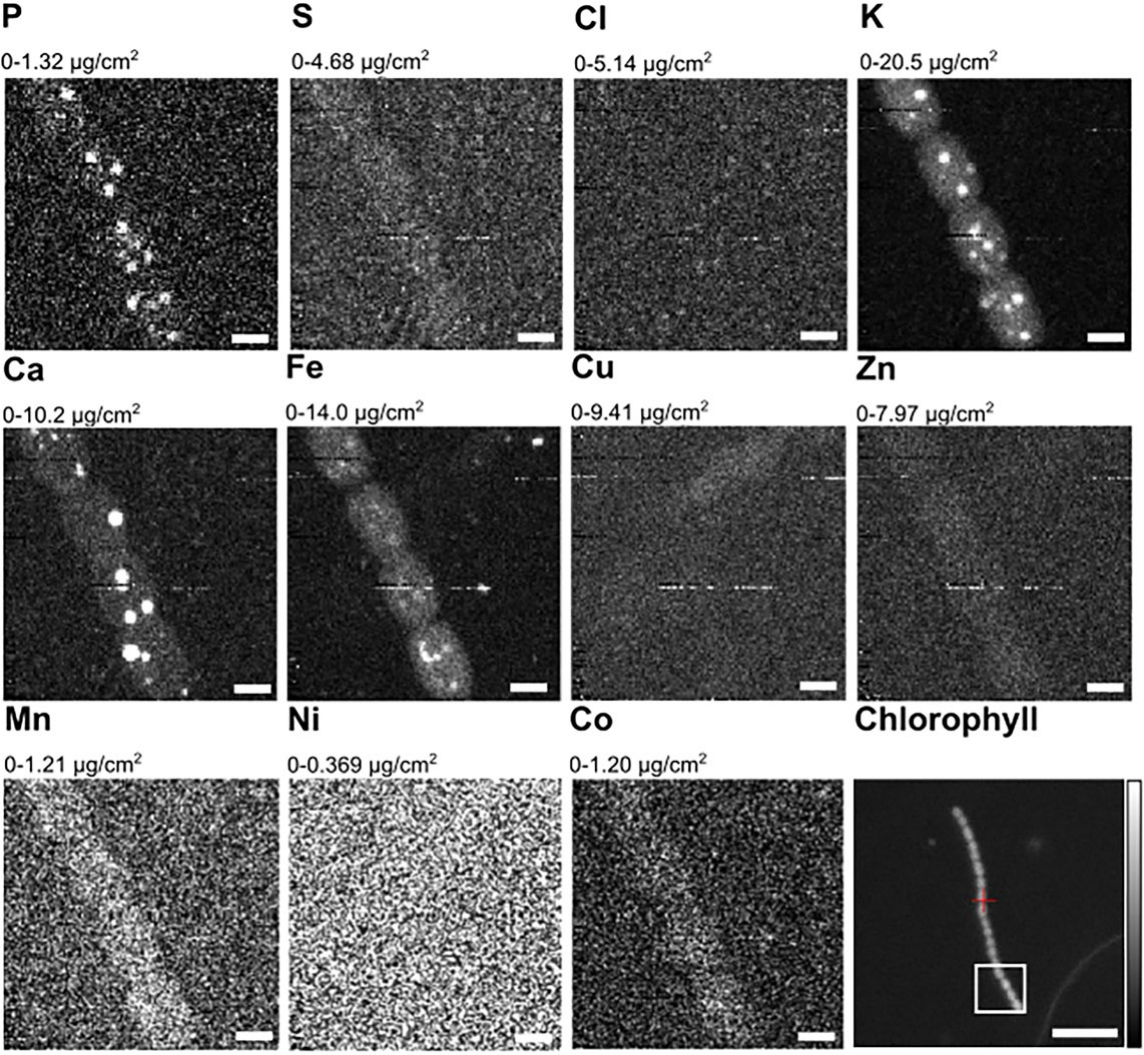
High-resolution X-ray fluorescence mapping of filamentous *Anabaena* sp. showing four vegetative cells. Elemental distribution mappings of P, S, Cl, K, Ca, Fe, Cu, Zn, Mn, Ni, and Co are displayed. Scale bars equal 2 μm (XRF panels) and 20 μm (chlorophyll).

In 2D XRF mappings (Figs. 6 and S5), S was found to be homogeneously distributed across the cellular ultrastructure of *Anabaena* sp. without salient hotspots. While the overall high cytosolic S levels are probably attributed to inorganic SO_4_^2−^, S is also a critically important constituent of sulfolipid thylakoid membranes important for the light-dependent oxygenic photosynthesis reaction,^60^ iron metabolism,^61^ and molybdate transport in cyanobacteria.^14^

**Fig. 7:**
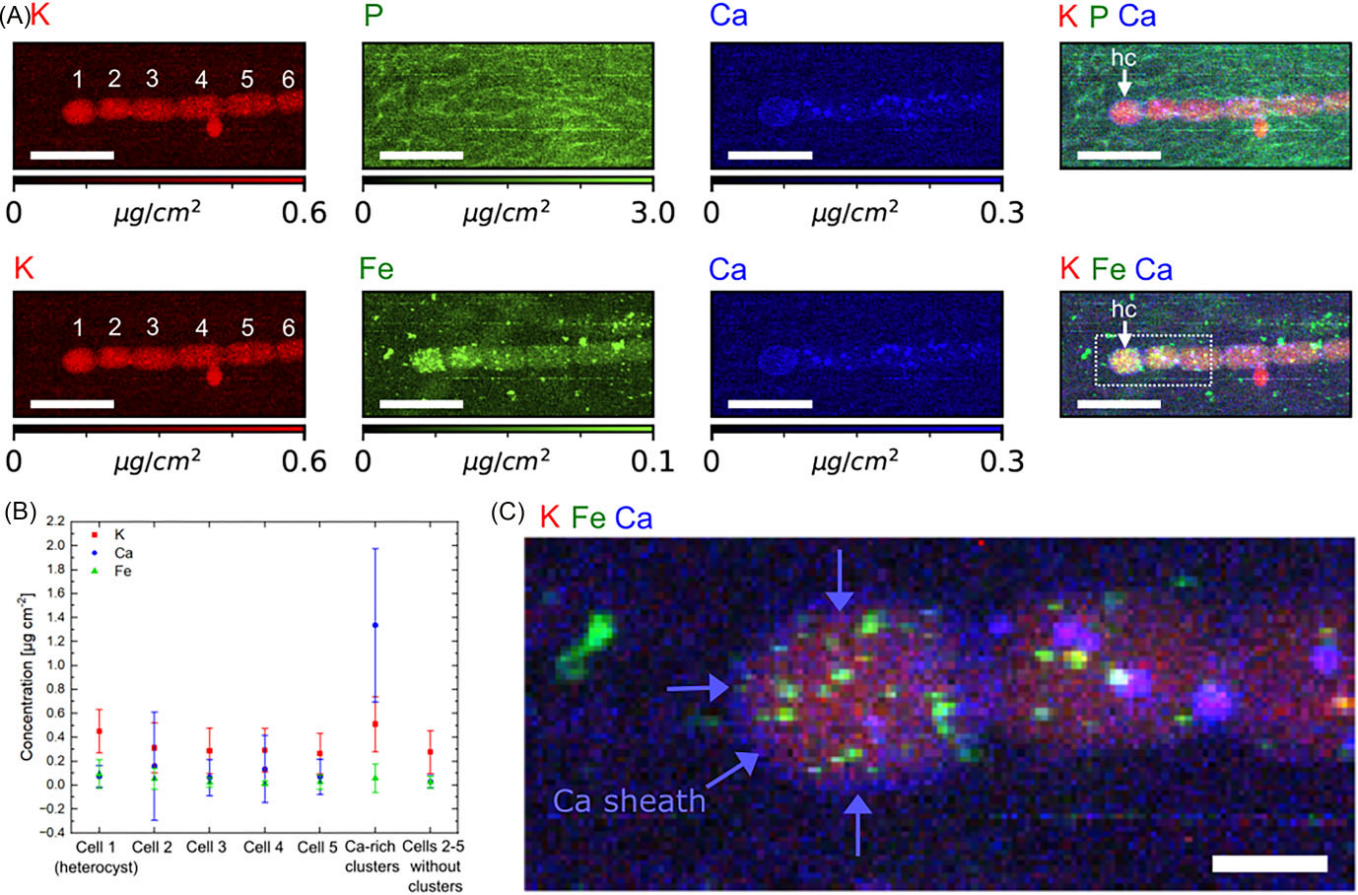
High-resolution X-ray fluorescence mapping of filamentous *Anabaena* sp. containing vegetative and heterocyst (hc) cells. (A) Elemental mappings of K, P, Ca, and Fe and their colocalization are displayed. (B) Elemental concentrations of K, Ca, and Fe in cell 1 (heterocyst), cells 2–5 (vegetative cells), Ca-rich clusters, and cytoplasm outside of clusters. Color bars were scaled to represent the 98th percentile of the concentration of each element. (C) Zoom-in mapping of K-FE-Ca elemental overlay. Scale bars equal 10 μm (A) and 2 μm (C).

Fe was found to be the most prevalent transition metal in *Anabaena* sp. filaments and key to understanding the enzymatic activity of nitrogen-fixing bacteria. Fe is involved in many biological functions in vegetative and heterocyst cells. In heterocysts, the enzyme for nitrogen fixation, nitrogenase, comprises three redox-active metal-containing cofactors including a [4Fe-4S] cluster, an eight-iron P cluster and a seven-iron plus molybdenum FeMo-cofactor.^14^,^24^,^25^,^62^,^63^ Ferric uptake regulator (Fur) proteins are global transcriptional regulators involved in Fe homeostasis of prokaryotes, which coordinate Fe^2+^ availability in the face of potential oxidative stress.^16^ Ferredoxins are iron–sulfur proteins that mediate electron transfer during photosynthesis.^21^ Fe limitation is therefore known to reduce the photosynthetic activity and cell growth of nitrogen-fixing cyanobacteria.^16^ Given the multifaceted role of Fe in cellular function, Fe was found throughout the cytosol as well as concentrated in irregularly shaped clusters in both cell types (Figs. 3 and 7). While some Fe clusters seem to be associated with polyP bodies and K^+^ clusters judging from 2D projections, other Fe clusters did not coincide with other elements. As expected, a three times higher concentration of Fe was detected in the heterocyst (0.095 ± 0.12 μg/cm^2^) than in neighboring vegetative cells of the same filament (0.027 ± 0.05 μg/cm^2^), as summarized in Fig. 7B and Table S2 in the supplementary information. Interestingly, a slightly higher Fe concentration was detected in the vegetative cell (0.054 ± 0.09 μg/cm^2^) next to the heterocyst, suggesting that the elevated micronutrient demands of the heterocyst are met by mobilizing cytosolic Fe^2+^ in neighboring cells. Our data imply that Fe clusters are characterized by a broad size dispersion with a mean area of 0.050 + 0.095 μm^2^ (Fig. 5).

Apart from Fe, other transition elements, such as Mn, Co, Ni, Cu, and Zn, present in the growth medium were probed with the Bionanoprobe (Figs. 6, S1 and S5). In cyanobacteria, trace metals fulfill important cellular functions as cofactors of metalloenzymes, specifically Cu in thylakoidal plastocyanin, Zn in carboxysomal carbonic anhydrase, Co in cobalamin, and Mn in the thylakoidal water-splitting oxygen-evolving complex.^64^ While we could not identify specific organelle structures, we found intracellular concentrations of Zn, Mn, and Co above background level. Ni, which is part of the [Ni-Fe] uptake hydrogenase enzyme that catalyzes the oxidation of H_2_ to recover the electrons released during nitrogen fixation,^65^,^66^ does not seem to be present in a high enough concentration to be detectable by XRF.

### X-ray fluorescence nanocomputed tomography

**Fig. 8:**
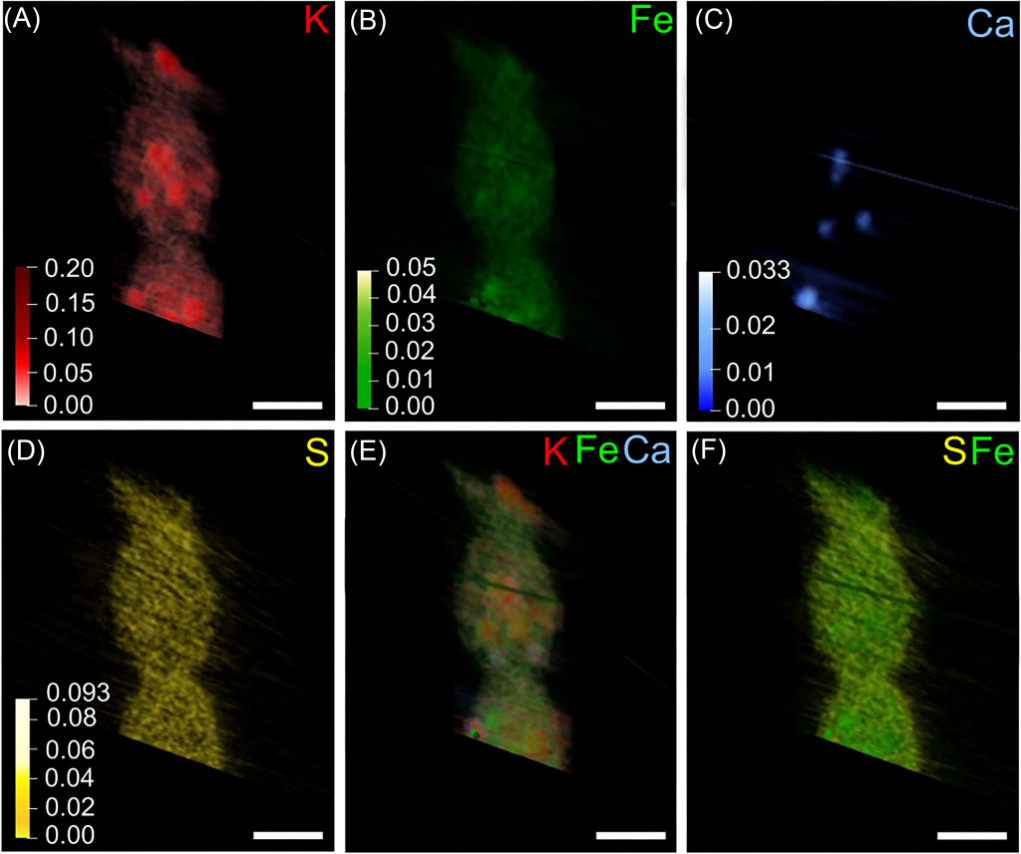
Three-dimensional X-ray fluorescence tomography of three adjacent vegetative cells. (A–D) The elemental signals of K, Fe, Ca, and S are displayed separately. (E) The overlay of K, Fe, and Ca 3D elemental distribution proofs that some elemental clusters a co-localized, while others are not. (F) The overlay of S and Fe elemental signals reveals that the outer cell envelope contains S, probably in the form of sulfolipids. Scale bars equal 2 μm.

The 3D arrangement and element makeup of three adjacent vegetative cells at the end of a long filament shown in Fig. 3 was studied by non-destructive X-ray fluorescence nanotomography (Figs. 8 and S9). Three-dimensional X-ray fluorescence imaging presents a powerful tool for structural and elemental imaging of frozen-hydrated biological specimens in a non-destructive manner. A unique advantage of freezing whole intact cells is the high preservation quality of the cellular ultrastructure.^67^,^68^ Apart from retaining subcellular structures in their original state, the cryogenic sample preservation imaging minimizes the loss of diffusible ions. A shortcoming of 2D XRF mappings is that whole bacteria cells measuring a few micrometers are viewed in a single projection. In tomography, we can elucidate whether two elements are truly co-localized in space and accurately evaluate their localization, dimension, and cluster shape. As shown in 2D XRF mappings, the elements K, S, and Fe are present throughout the cytoplasm. Due to imaging artefacts or the overall lower concentration, P was not included in the tomographic representation. K clusters appear oval to roundish, whereas Fe hotspots are comparably undefined in shape. The concentration of P was too low to be reconstructed in the 3D tomography. In the 3D representation, Ca clusters appear ellipsoidal when viewed from multiple angles (Fig. S10; video 2). While K and Fe clusters are found in various areas, it is evident that all four Ca clusters are located in the cell periphery (Fig. S10; video 2), which could be related to their association with RuBisCO-storing microcompartments. When zooming in, it appears that Ca is forming a layer around K-containing hotspots (Fig. S10). Based on 3D data, it is evident that some of the Fe and Ca clusters are not co-localized with any of the other abundant elements (Fig. 3), which means that they are unlikely to be sequestered by polyP bodies. The 3D tomography further reveals that S is homogeneously spread across the cytosol, but in contrast to Fe, K, and Ca, which are confined to the interior cell, it seems to be part of sulfolipids found in the outer cell envelope forming the periplasmatic space connecting multiple cells.

### Energy-dispersive X-ray imaging in the Scanning TEM

**Fig. 9:**
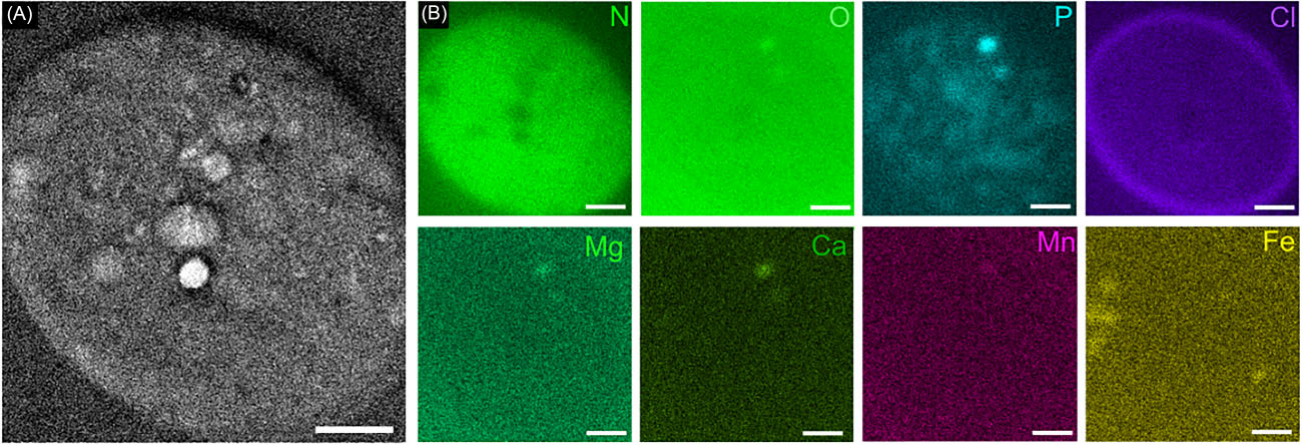
STEM-DF micrograph of unstained, embedded, and 200-nm thin-sectioned vegetative cell showing granular features in cytosol and gas vacuoles. Scale bar equals 1 μm. (B) Energy-dispersive X-ray spectroscopic mappings provide semi-quantitative elemental distribution of N, O, P, Cl, Mg, Ca, Mn, and Fe. Scale bar corresponds to 500 nm.

Using Scanning Transmission Electron Microscopy (STEM), we detected electron-dense granules and gas vacuoles in unstained samples. To study the subcellular distribution of lighter elements (Z < 13), which could not be examined with XRF at the given X-ray energy, we employed energy-dispersive X-ray microanalysis in STEM. Thin sections were prepared by epoxy embedding and room-temperature ultramicrotomy. According to STEM-EDS mappings, N is homogenously distributed across the cell, whereas P reflects the granular texture of the cytoplasm. P-rich granules are likely to be associated with previously observed polyP, which are enriched in oxygen, and accumulate monovalent and divalent cations, such as K^+^, Ca^2+^, and Mg^2+^ and the transition elements Fe^2+^ and Mn^2+^. Mg fulfills multiple cellular functions, e.g. as part of light-harvesting chlorophyll in the thylakoid membranes^69^ or by controlling the circadian rhythm.^70^ Intriguingly, we observe higher levels of Cl^−^ in the outer sheath of the cell. It is possible that the ∼345 ± 41 nm thick Cl-containing sheath was not properly resolved in 2D XRF mappings of 100 nm pixel size. Notably, the thickness of this Cl-rich layer exceeded the previously reported dimension of the ∼100 nm thick, multilayered cyanobacterial cell envelope.^71^ STEM-EDS data (Figs. 9 and  S11) confirm that Fe appears in separate hotspots not associated with polyP granules, which might be related to its multifaceted enzymatic activity. While STEM/EDS results are generally in good agreement with XRF mappings from frozen-hydrated samples, it is possible that the invasive sample preparation procedure, including dehydration and resin embedding necessary for conventional STEM/EDS data collection, resulted in a loss or redistribution of water-soluble ions.

## Conclusions

Over millions of years, nitrogen-fixing cyanobacteria have played a vital role in the Earth's biogeochemical carbon and nitrogen cycle. To date, nitrogen-fixing cyanobacteria are an integral part of freshwater, marine, and terrestrial ecosystems, surviving even in extremely harsh habitats, such as hot springs or saline alkaline lakes.^72^ Cyanobacteria, also called blue green algae, form thick harmful algae blooms, contributing to the eutrophication of oceans, lakes, and rivers, releasing toxins into the environment, and compromising potable water supplies. Future studies involving a systematic variation of ions in the culture medium or field sampling from natural waters will shed light on environmental factors favoring nitrogen fixation and influencing complex microbial population dynamics. In the present study, a multimodal imaging approach provided in-depth structural and quantitative chemical information that advances our understanding of the effect of micronutrients on biological nitrogen fixation. P-rich clusters, presumably polyP bodies, occurred together with K^+^ and occasionally accumulated divalent cations, such as Ca^2+^, Mg^2+^, Fe^2+^, and Mn^2+^, suggesting a crucial role in maintaining trace metal homeostasis, nutrient storage, and osmosis regulation. According to XRF nanotomography, vegetative cells contain Ca clusters that enclose smaller P/K clusters close to the cyanobacterial cell wall. Heterocysts exhibited higher cytosolic levels of Fe, K, and Ca than vegetative cells, reflecting increased micronutrient demands because of nitrogen fixation. The heterocyst's outer exo-polysaccharide layer seems to be enriched in Ca^2+^, while Cl was found in the outer layer of vegetative cells. Taken together, visualizing the elemental distribution across the cellular ultrastructure of nitrogen-fixing cyanobacteria helps us establish a better understanding of the influence of micronutrients on the cell physiology under fixed nitrogen limitation. XRF measurements of frozen-hydrated cells under cryogenic conditions successfully preserved the integrity of samples, minimized X-ray beam damage, and prevented loss of light elements. This paper describes the first results obtained with a combined inline optical and X-ray fluorescence set-up at the Bionanoprobe. By marking specific organelles (e.g. carboxysomes) with fluorescent probes or by performing immunolocalization experiments, correlative optical fluorescence and X-ray fluorescence imaging open exciting experimental avenues in environmental biochemistry and cellular biology. Apart from resolving the elemental distribution with high spatial resolution and sensitivity, future synchrotron-based research may focus on elucidating the biological redox chemistry by specifying the oxidation state and chemical environment of metal(loid)s within biological environmental samples using X-ray absorption near-edge structure (XANES).

Diazotrophic cyanobacteria harbor tremendous possibilities as biotechnological platforms for producing basic chemicals. Thus, detailed information of the micronutrient requirements of nitrogen-fixing bacteria may promote the use of aquatic microorganisms as renewable resources and green energy producers. The reduction of carbon dioxide emissions is imperative to halt climate change and prevent detrimental environmental changes. Photosynthetic microorganisms present an effective CO_2_-sink, but while plants require an external source of nitrogen for optimal growth, specific strains of cyanobacteria are capable of fixing nitrogen from air. Some cyanobacteria produce hydrocarbons (e.g. pentadecane) that can be directly used as biofuels without the need for transesterification.^73^,^74^ A better understanding of the enzymatic activity in cyanobacteria may aid the development of synthetic catalysts for the energy-efficient production of ammonia and hydrogen.^62^,^63^,^75^ At present, the industrial conversion of nitrogen to ammonia alone consumes more than 1% of the global energy production.^75^,^76^ Ammonia is widely used as fertilizer or base chemical for many intermediate products. In contrast to the industrial production of ammonia, the enzymatic pathway yields at least one equivalent of molecular hydrogen,^62^ which has multiple industrial applications, for instance, in fuel cells. Alternatively, nitrogen-fixing cyanobacteria can be directly employed as biological fertilizers for rice cultivation, effectively reducing eutrophication^73^,^74^,^77^ or for wastewater bioremediation.^78^

## Supplementary Material

mfae040_Supplemental_Files

## Data Availability

Data will be made available on request.
