# Supplementary material for: Nanoscale elemental and morphological imaging of nitrogen-fixing cyanobacteria
Source: Metallomics. 2024 Sep 13;16(10):mfae040. doi: 10.1093/mtomcs/mfae040 (PMC11450467; doi:10.1093/mtomcs/mfae040)
Supplement: mfae040_Supplemental_Files [file mfae040_supplemental_files.zip › Suppl_data_Supporting_info_Nanoscale_elemental_and_morphological_imaging_of_nitrogen-fixing_cyanobacteria_FINAL.docx]

**Supplementary material**

The Supporting Information is available free of charge (PDF). Figures show 2D and 3D X-ray fluorescence mappings, elemental colocalization mappings, cluster size distributions, and STEM-EDS micrographs. Tables 1 and 2 give quantitative elemental concentrations. Videos 1 and 2 show XRF nanotomography of Fe and K/Ca.


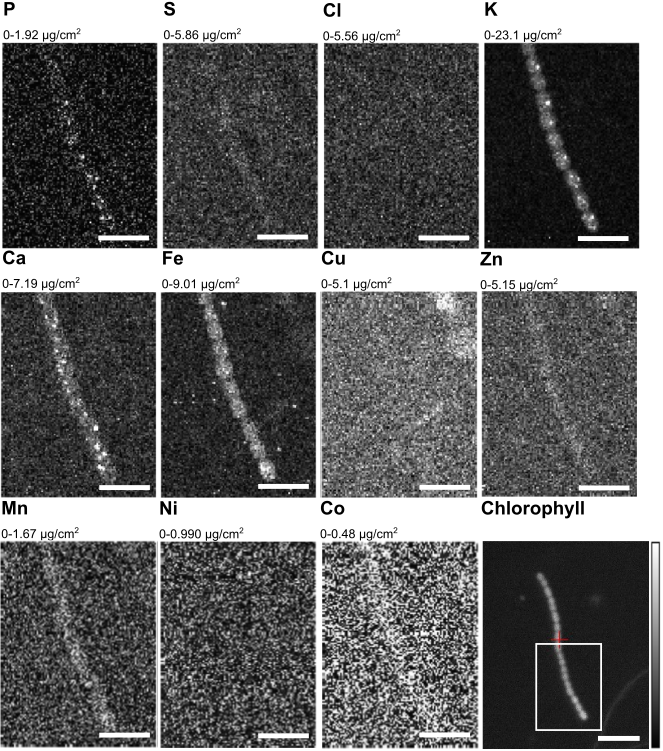


**Figure S1.** High-resolution X-ray fluorescence mapping of filamentous Anabaena sp. containing long chain of vegetative cells. Elemental distribution mappings of P, S, Cl, K, Ca, Fe, Cu, Zn, Mn, Ni, and Co are displayed. A narrower color range was applied to enhance contrast of trace elements. Scalebar equals 10 μm (elements) and 20 μm (chlorophyll).


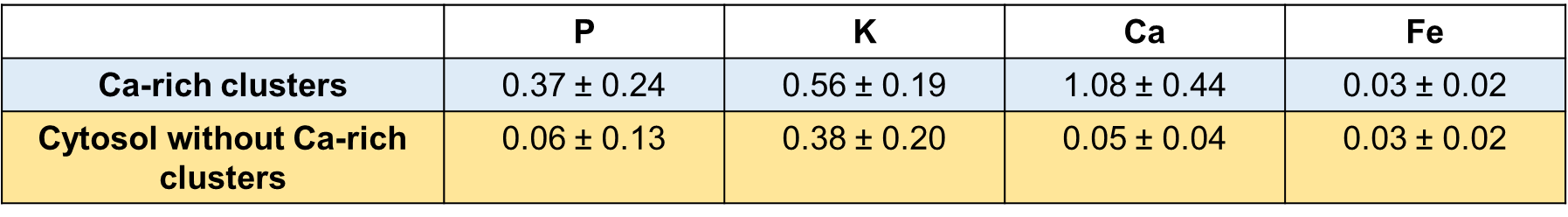

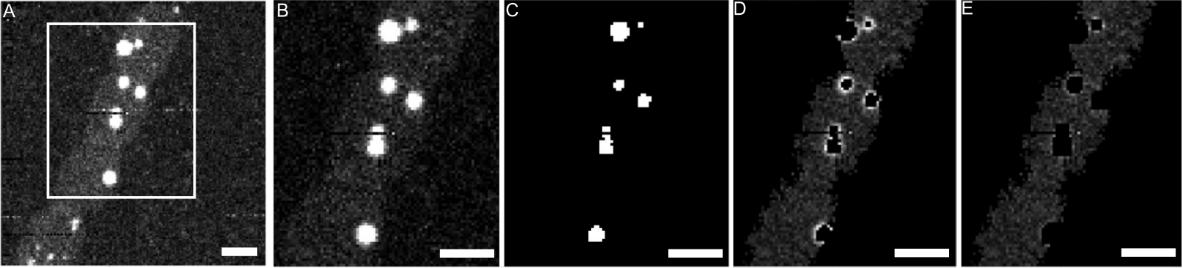


**Table S1.** Average concentration (μg/cm^2^) of cytosol and clusters after background subtraction from Figure 3. Standard deviation is calculated for all the pixels in the image.

**Figure S2.** High-resolution X-ray fluorescence mapping of filamentous Anabaena sp. showing four vegetative cells. Elemental distribution mappings of P, S, Cl, K, Ca, Fe, Cu, Zn, Mn, Ni, and Co are displayed. Scalebar equals 2 μm.


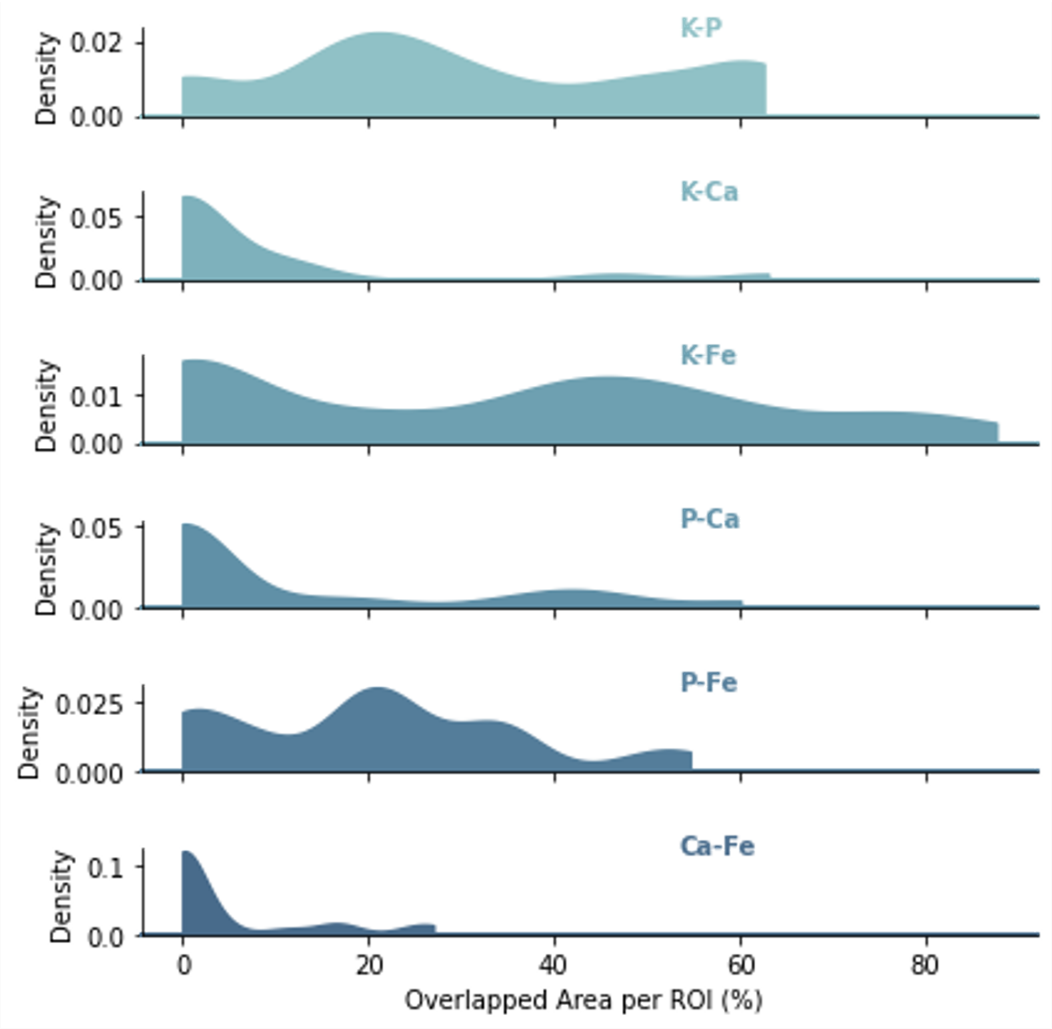


**Figure S3.** Overlap area per ROI for different pairs of elements in Figure 3.


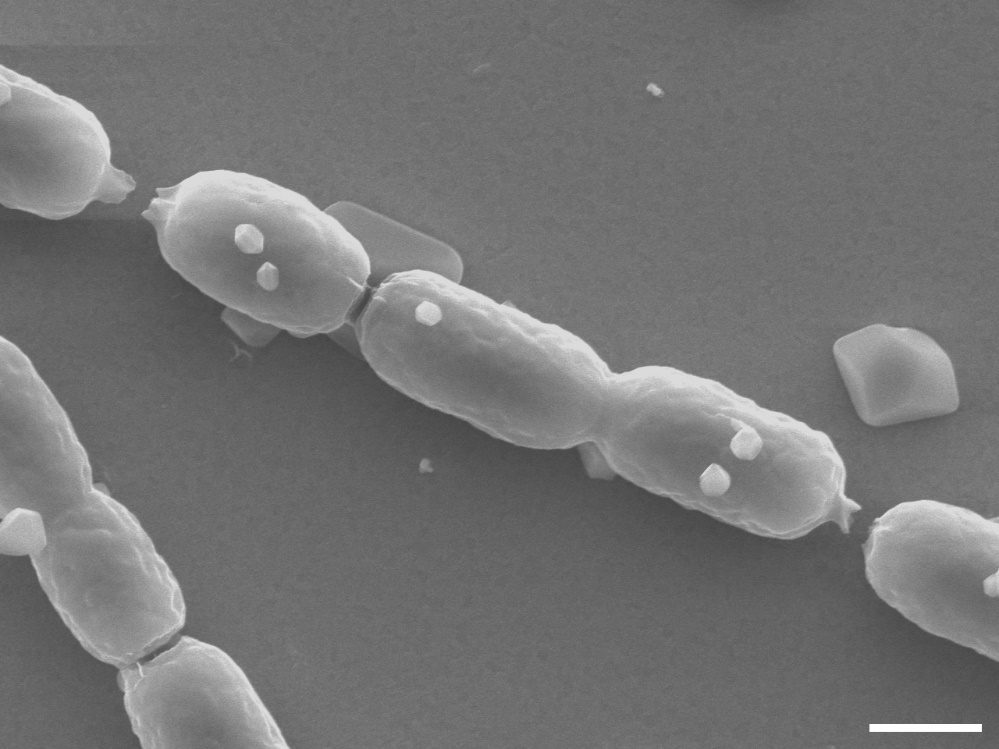

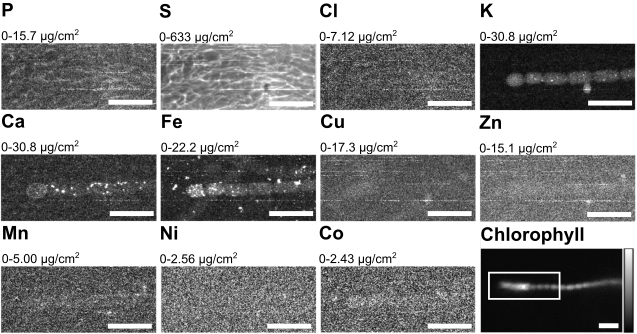


**Figure S4.** FE-SEM from Anabaena sp. filaments, presumably with inorganic crystallites. Scalebar equals 2 μm.

**Figure S5.** High-resolution X-ray fluorescence mapping of filamentous Anabaena sp. containing vegetative and heterocyst (hc) cells. Elemental distribution mappings of P, S, Cl, K, Ca, Fe, Cu, Zn, Mn, Ni, and Co are displayed. Scalebars equal 10 μm.


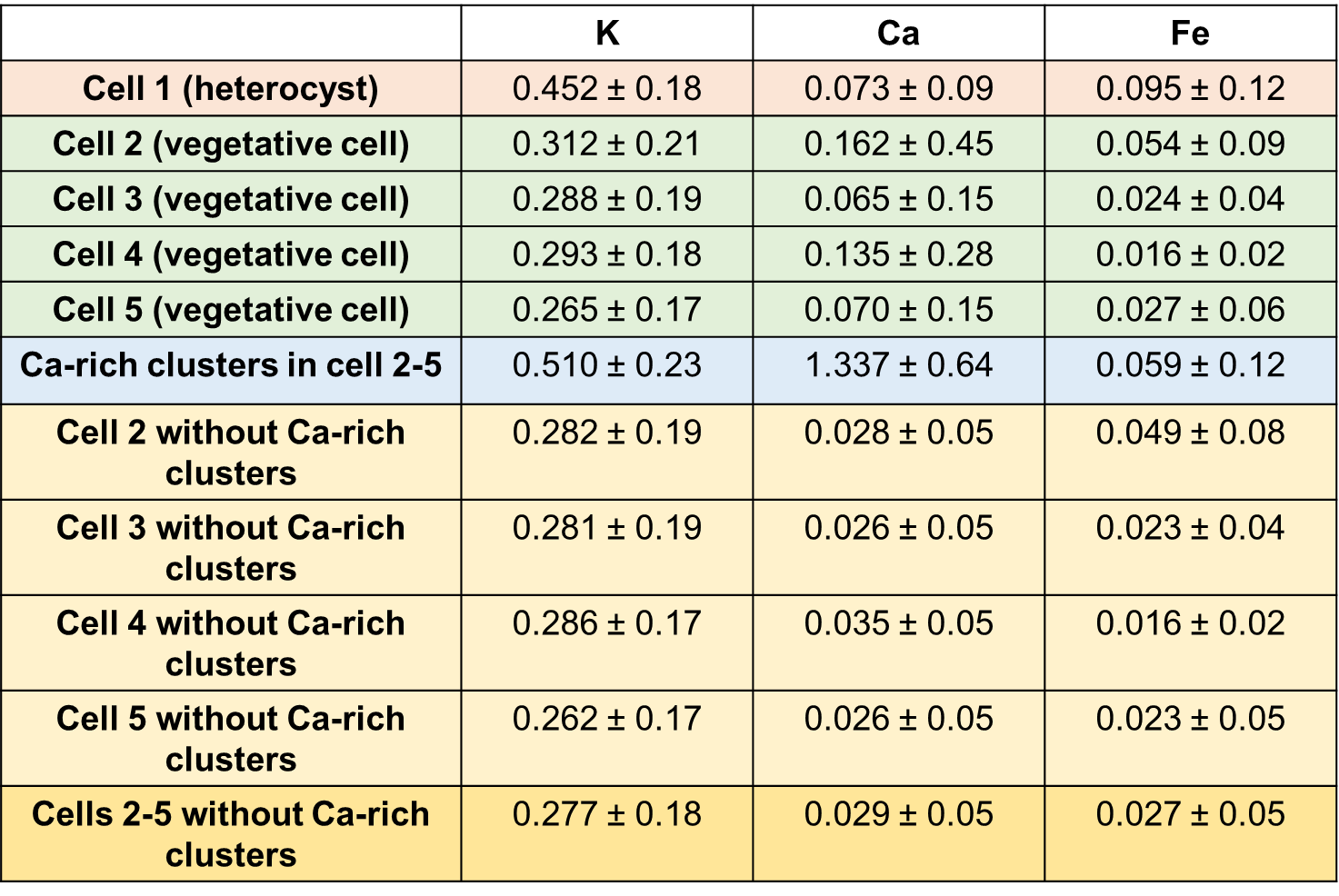


**Table S2.** Average concentration (μg/cm^2^) of cytosol and Ca-rich clusters after background subtraction from Figure 4. P was not quantified due to high fluctuating background levels in this mapping. Standard deviation is calculated for all the pixels in the image.

***
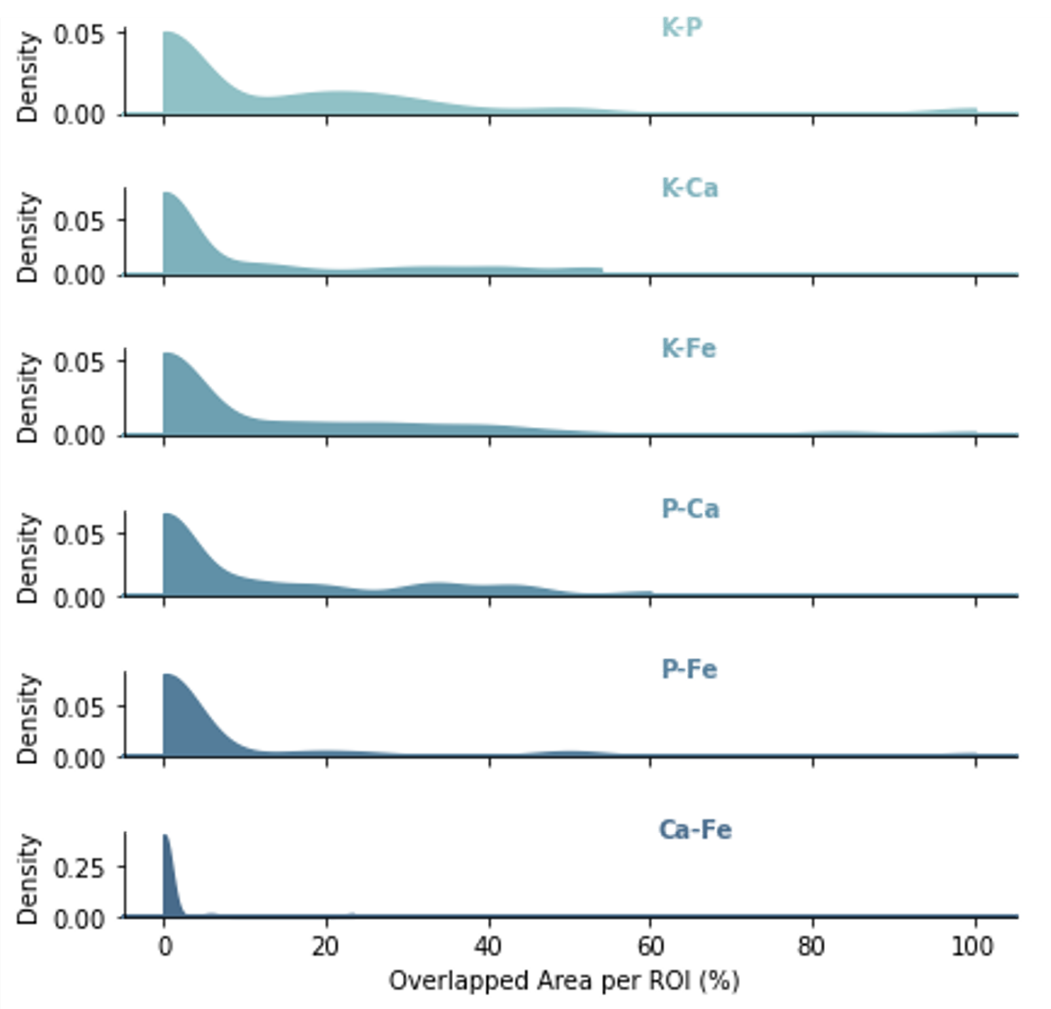
***

**Figure S6.** Overlap area per ROI for different pairs of elements in Figure 7.


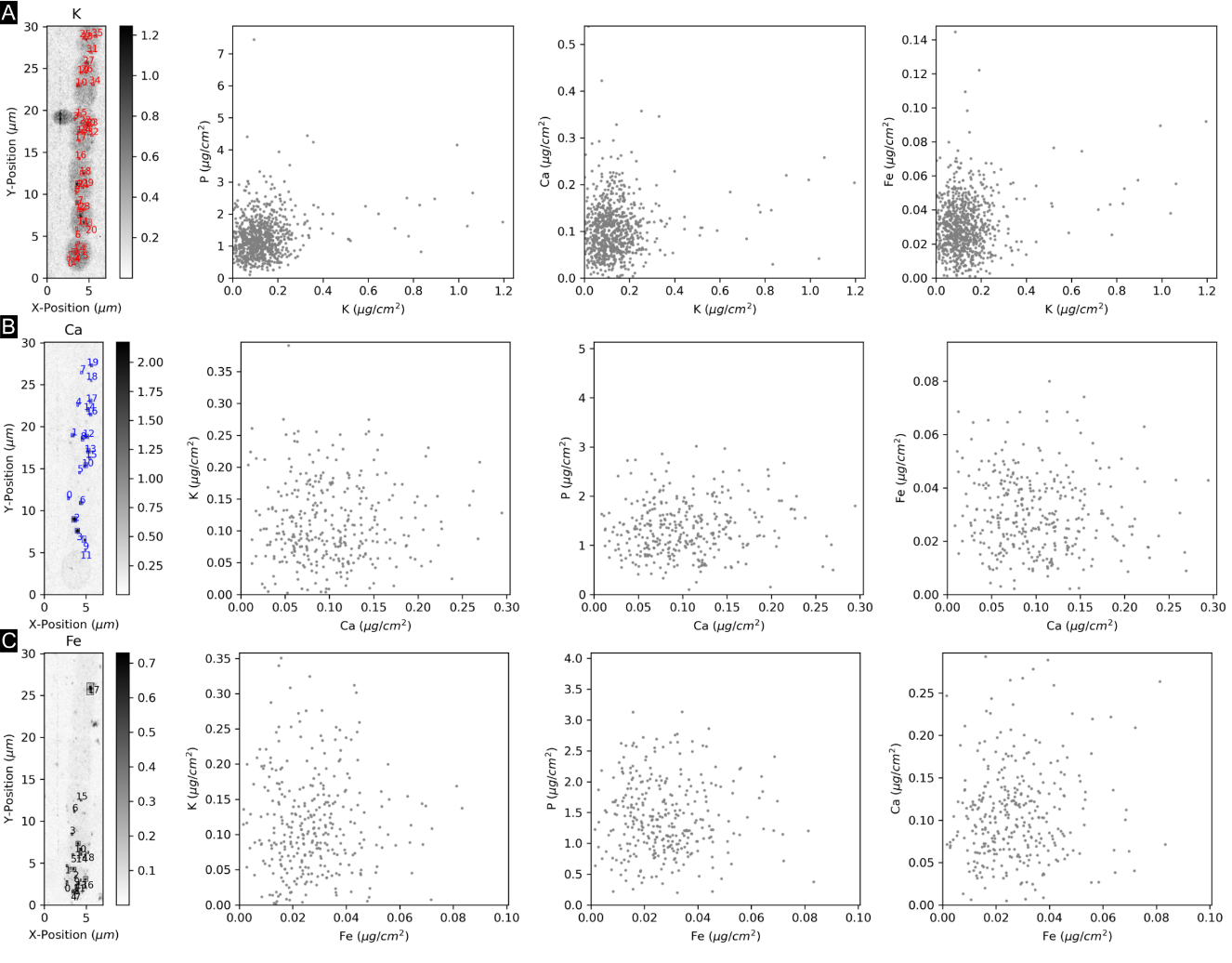

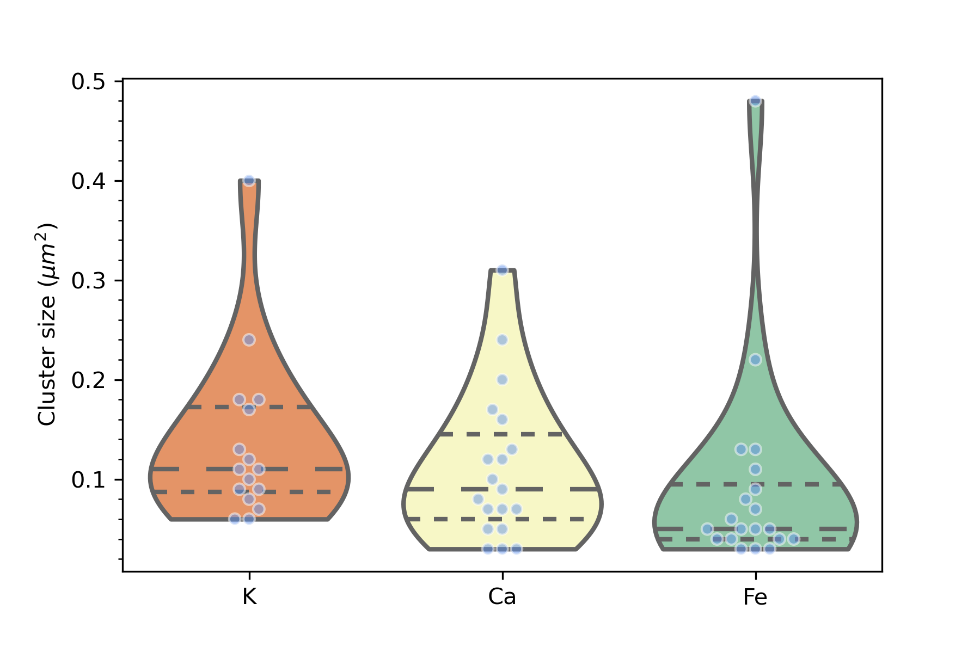


**Figure S8.** Cluster sizes for K, P, Ca and Fe elements detected within bounding boxes of Figures 7 were plotted as a violin plot. The central dashed line denotes the median. The three dashed lines within each shaded block show the 25th, 50th and 75th percentile of the data from bottom to top.

**Figure S7.** Machine learning based image segmentation and cluster analysis of XRF mapping shown in Figure 6. The bounding boxes detected around individual clusters vary in size and position, depending on the XRF signal of each element. Region of interest (ROI) analysis was based on elemental intensity with Pearson’s R-value. Color bars were scaled to represent the 99^th^ percentile of the concentration of each element.


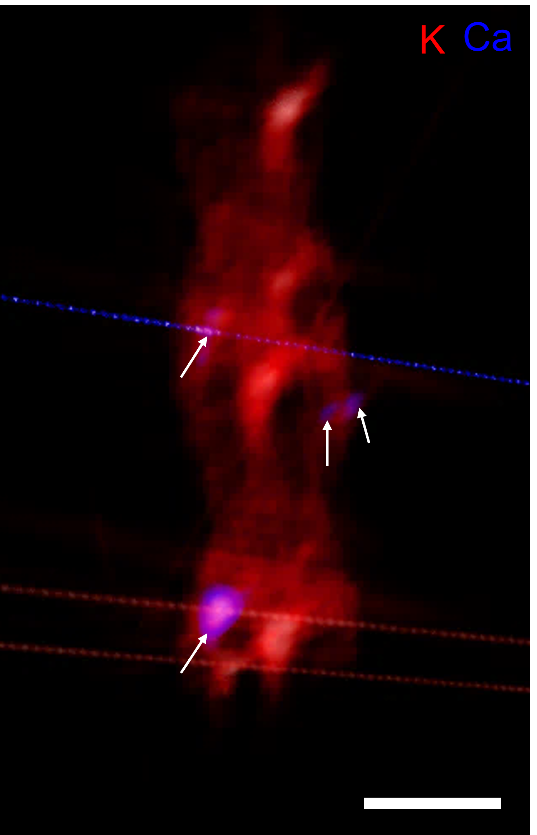

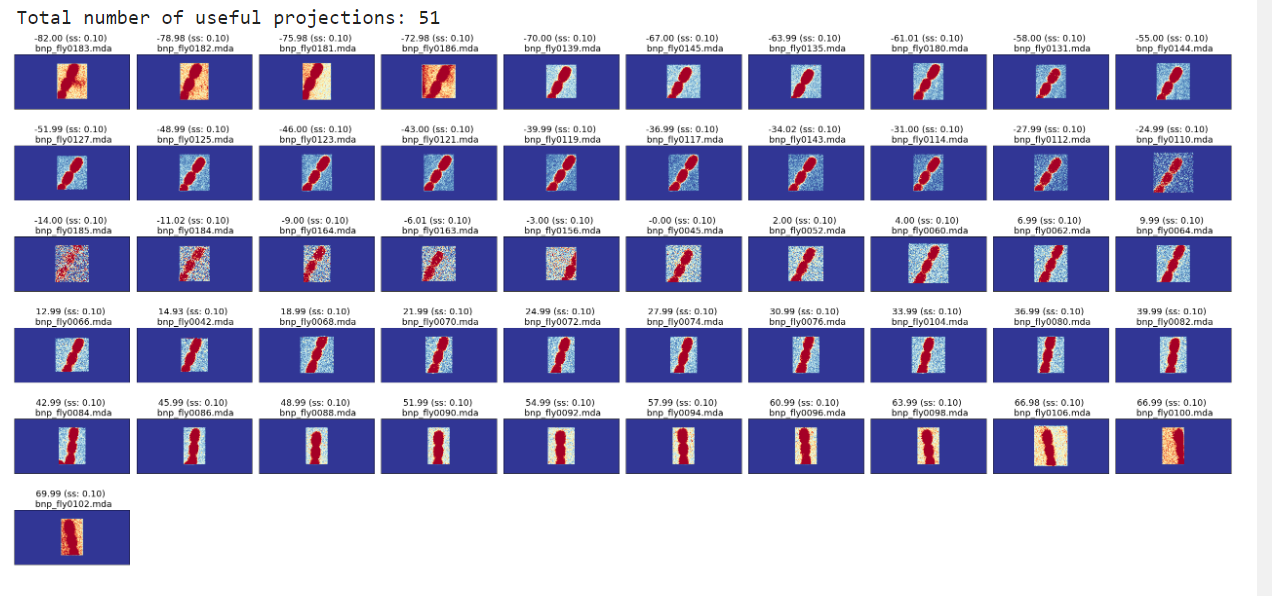


**Figure S10.** Three-dimensional X-ray fluorescence tomography of three adjacent vegetative cells, as shown in Figure 8. Ca/K clusters marked with arrows were found in the cell periphery. Ca (blue) is forming a sheath around K clusters (red). Scalebar equals 2μm.

**Figure S9.** XRF nanotomography was reconstructed from 51 projections with 100nm step size and 150 ms/pixel dwell time over an angular range of -82°-70°.


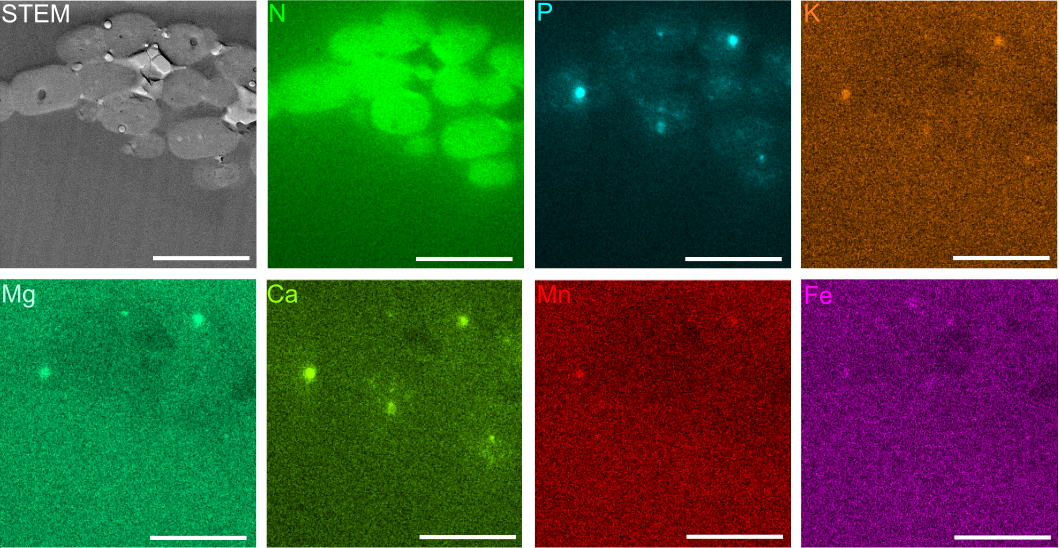


**Figure S11.** STEM micrograph of unstained, embedded, and 200nm thin-sectioned vegetative cells. Energy-dispersive X-ray spectroscopic mappings provide semi-quantitative elemental distribution of N, O, P, Mg, Ca, Mn, and Fe. Scalebar corresponds to 500 nm.
