# Supplementary figures and images for: Nanoscale elemental and morphological imaging of nitrogen-fixing cyanobacteria
Source: Metallomics. 2024 Sep 13;16(10):mfae040. doi: 10.1093/mtomcs/mfae040 (PMC11450467; doi:10.1093/mtomcs/mfae040)

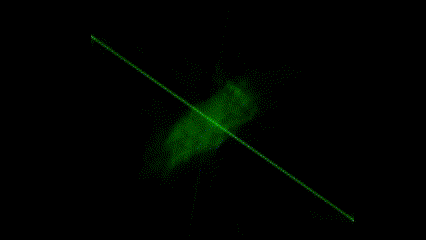

Supplement: mfae040_Supplemental_Files [file mfae040_supplemental_files.zip › Suppl_data_Fe_video.gif]

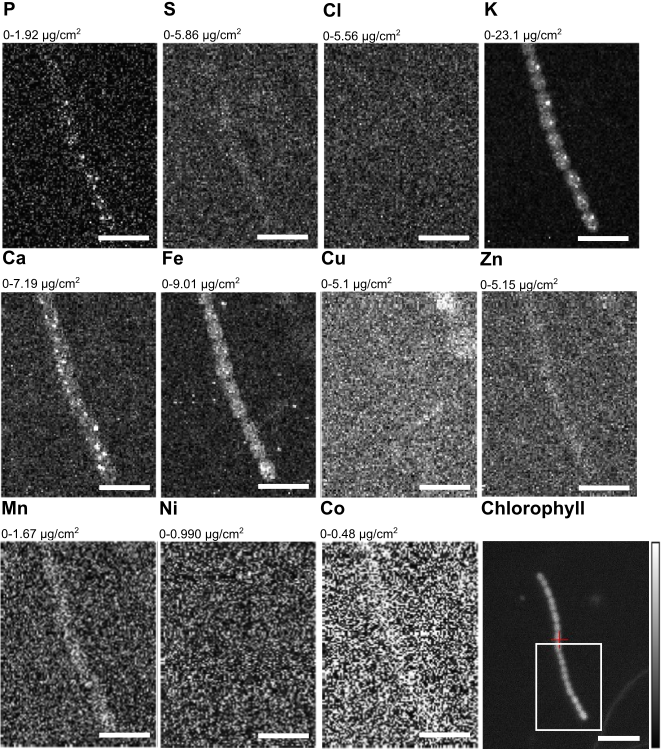

Supplement: mfae040_Supplemental_Files [file mfae040_supplemental_files.zip › Suppl_data_Figure_S1.tiff]

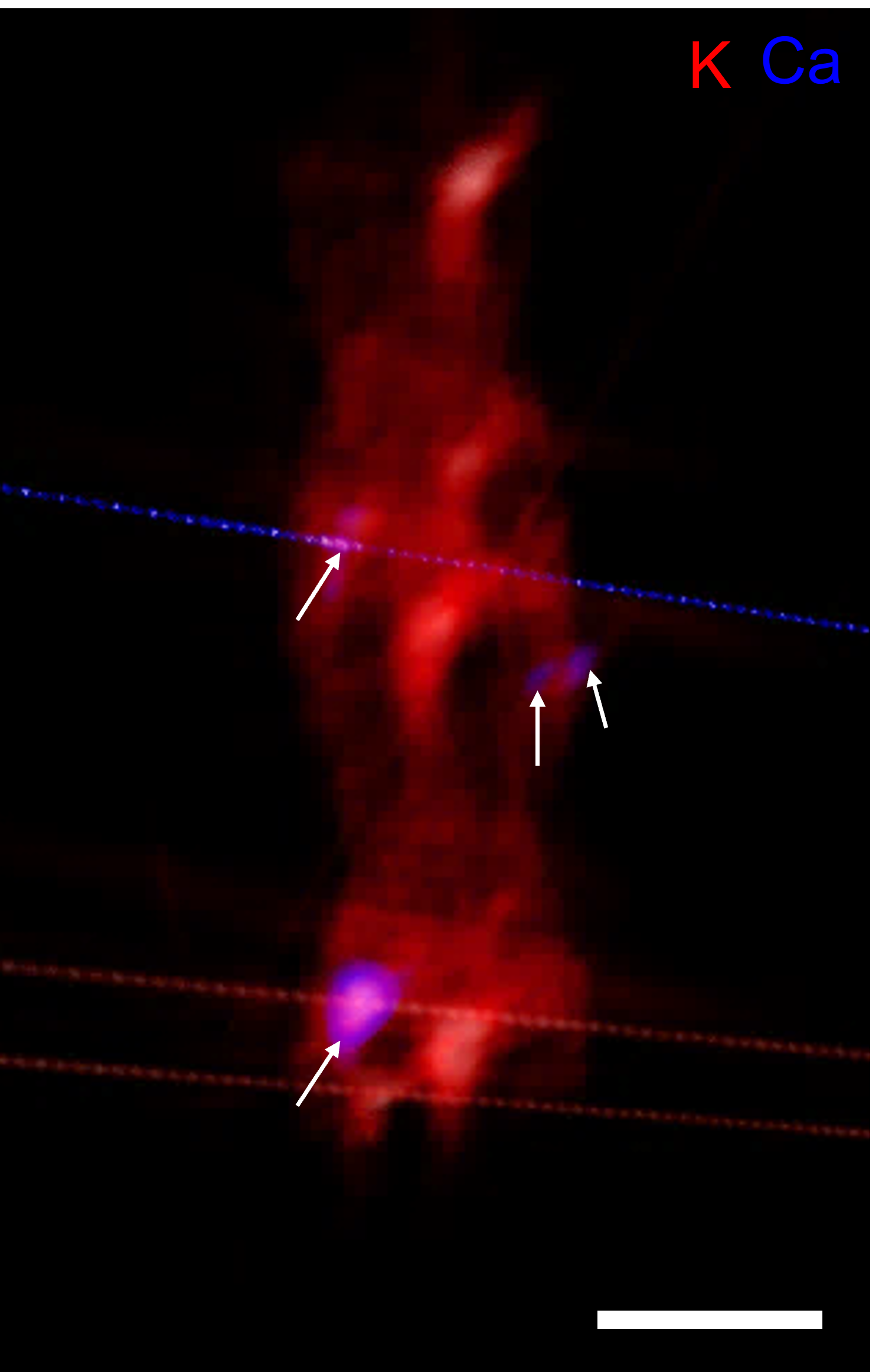

Supplement: mfae040_Supplemental_Files [file mfae040_supplemental_files.zip › Suppl_data_Figure_S10.tif]

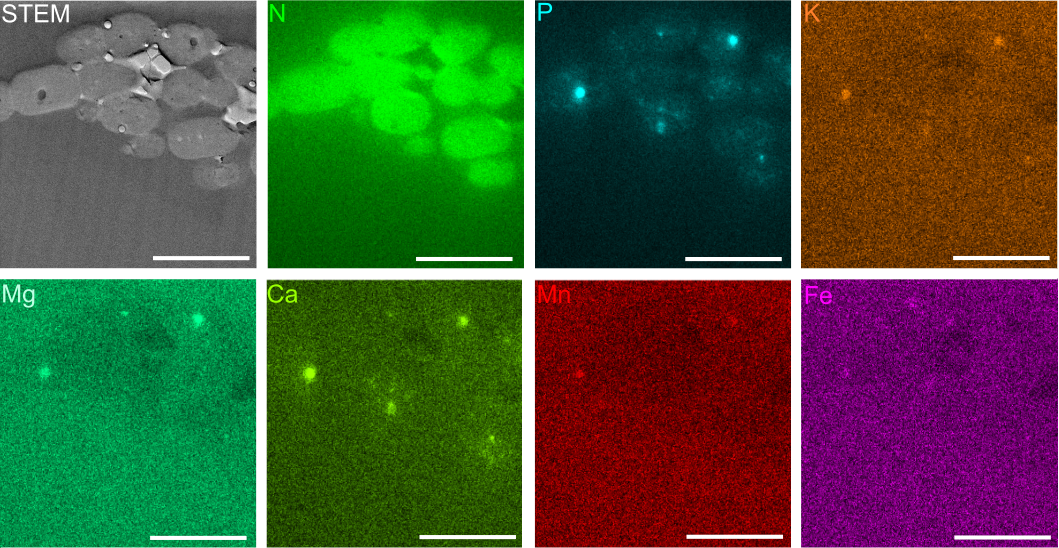

Supplement: mfae040_Supplemental_Files [file mfae040_supplemental_files.zip › Suppl_data_Figure_S11.tif]

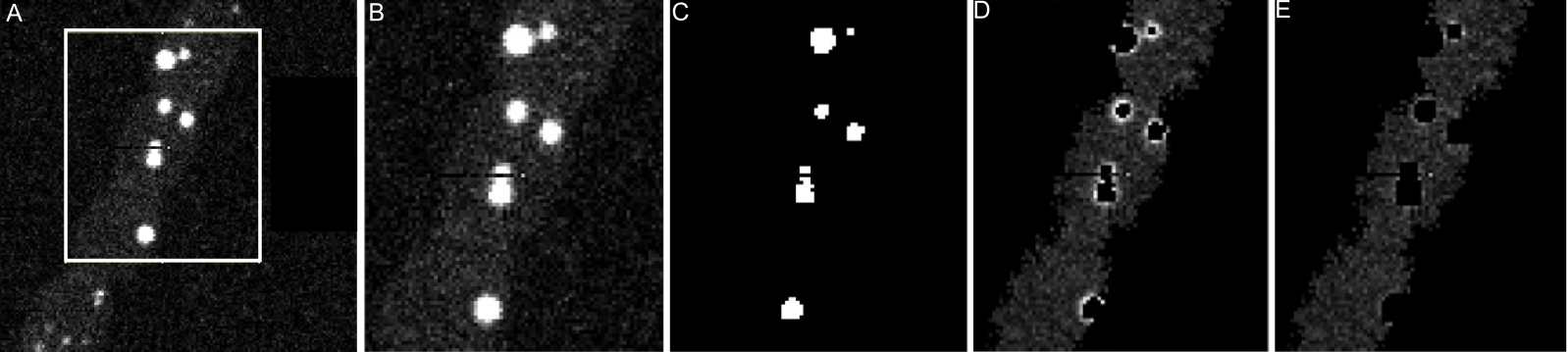

Supplement: mfae040_Supplemental_Files [file mfae040_supplemental_files.zip › Suppl_data_Figure_S2.tif]

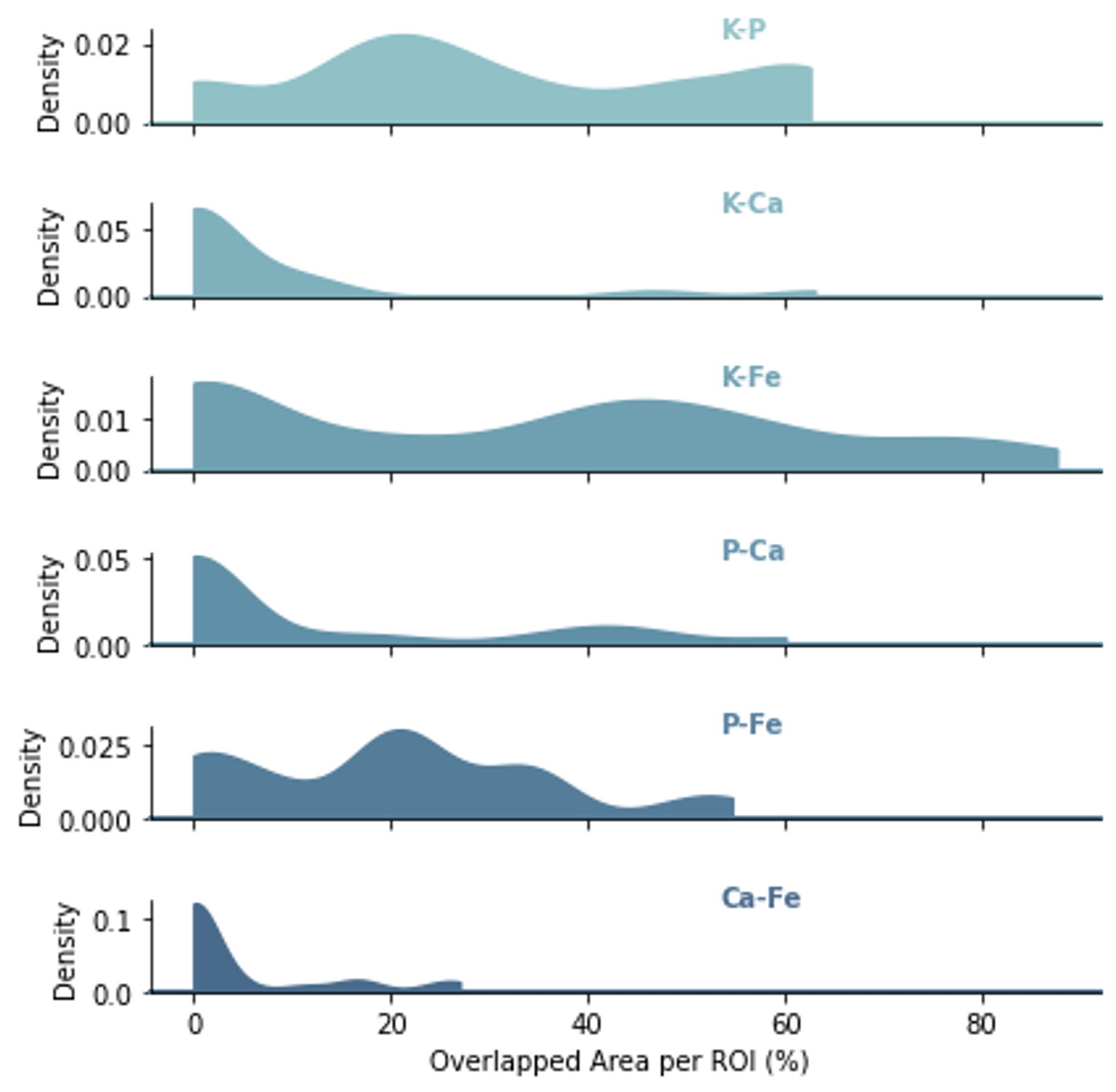

Supplement: mfae040_Supplemental_Files [file mfae040_supplemental_files.zip › Suppl_data_Figure_S3.tif]

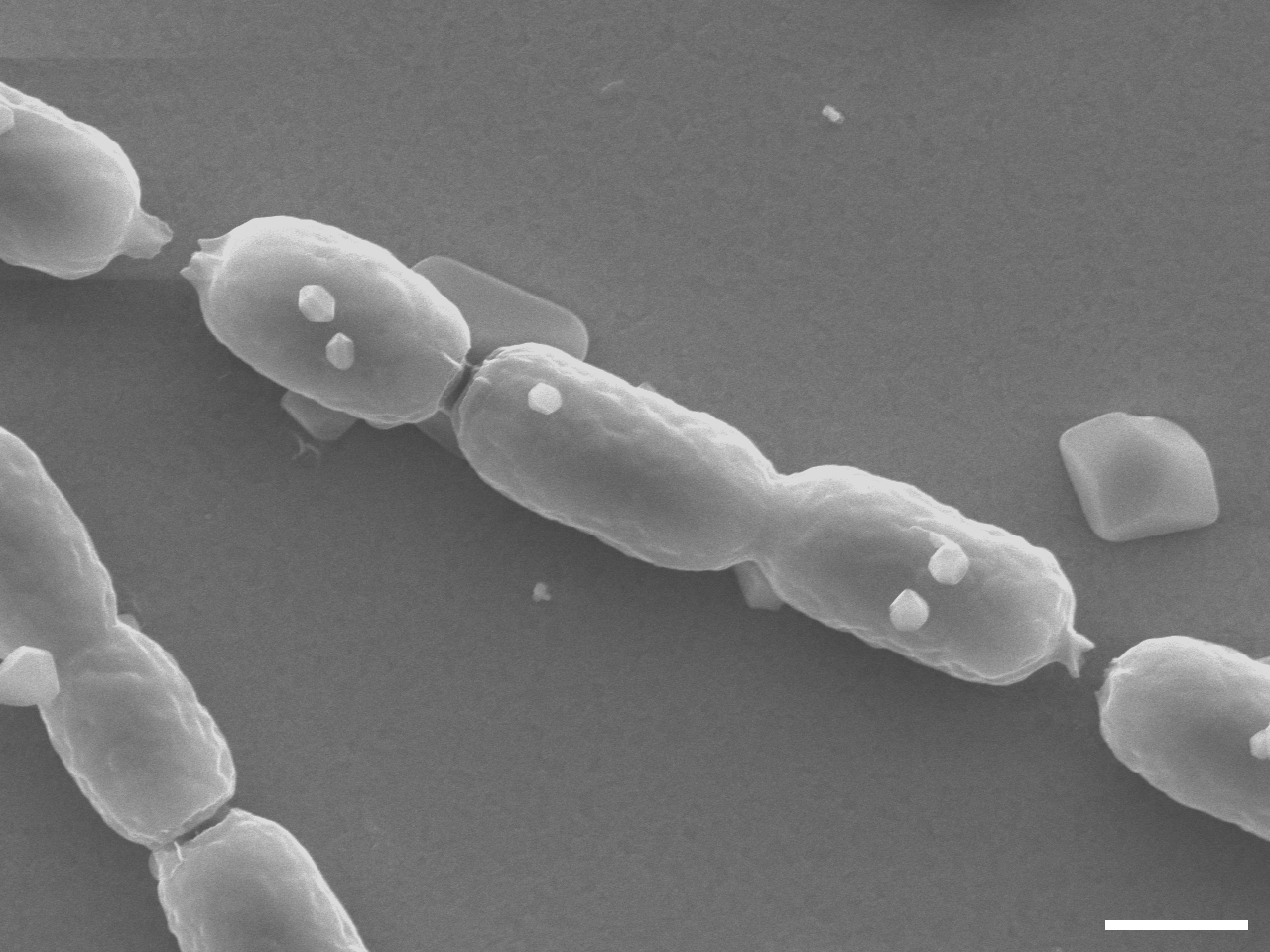

Supplement: mfae040_Supplemental_Files [file mfae040_supplemental_files.zip › Suppl_data_Figure_S4.tif]

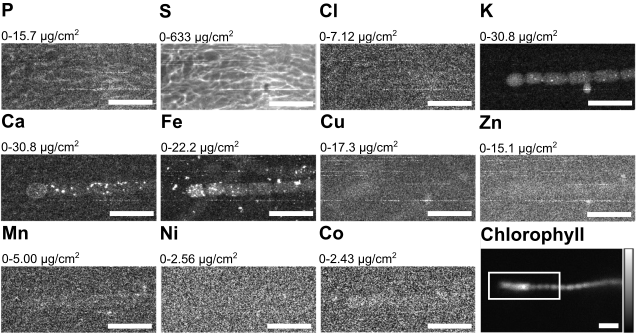

Supplement: mfae040_Supplemental_Files [file mfae040_supplemental_files.zip › Suppl_data_Figure_S5.tiff]

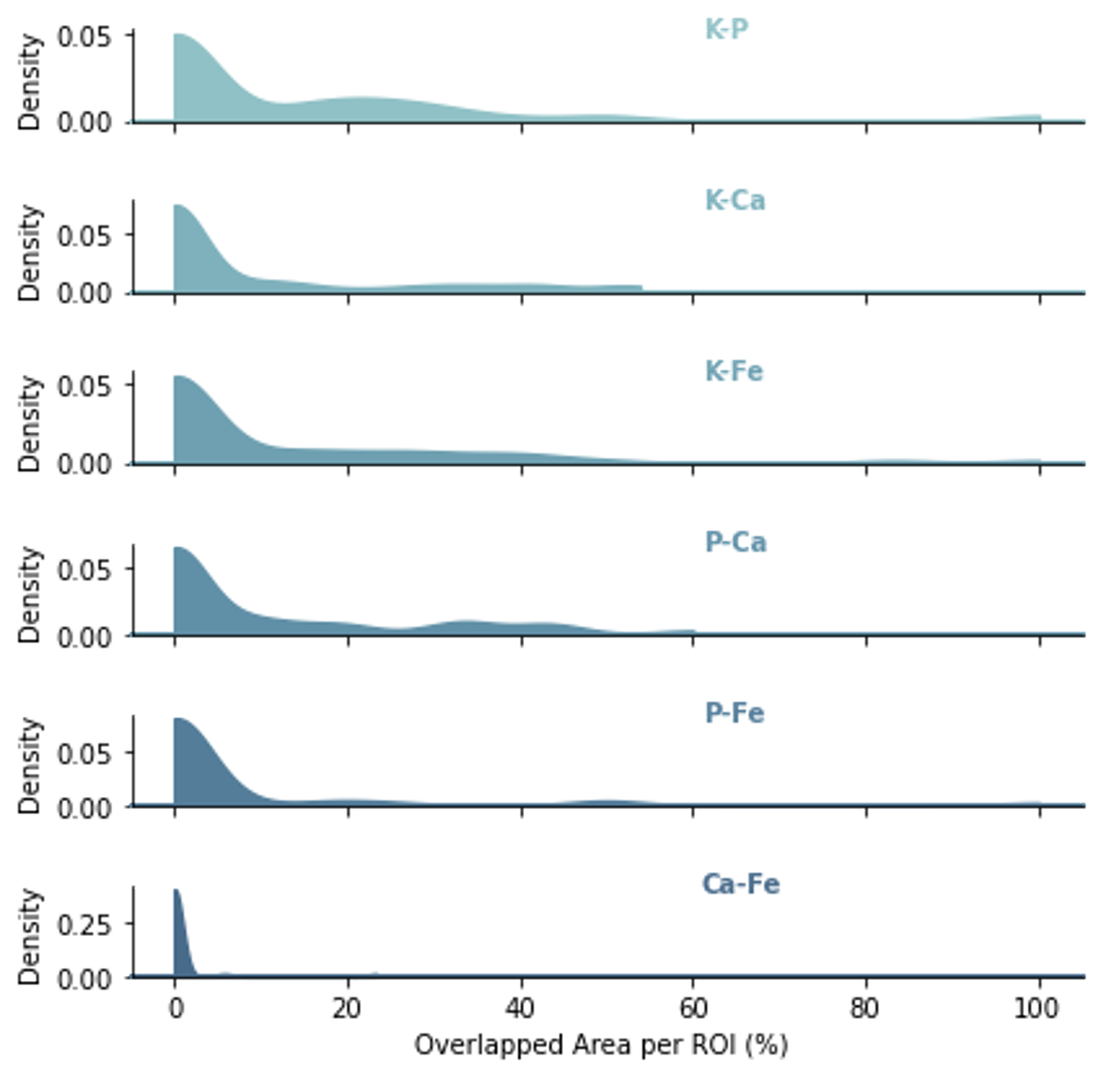

Supplement: mfae040_Supplemental_Files [file mfae040_supplemental_files.zip › Suppl_data_Figure_S6.tif]

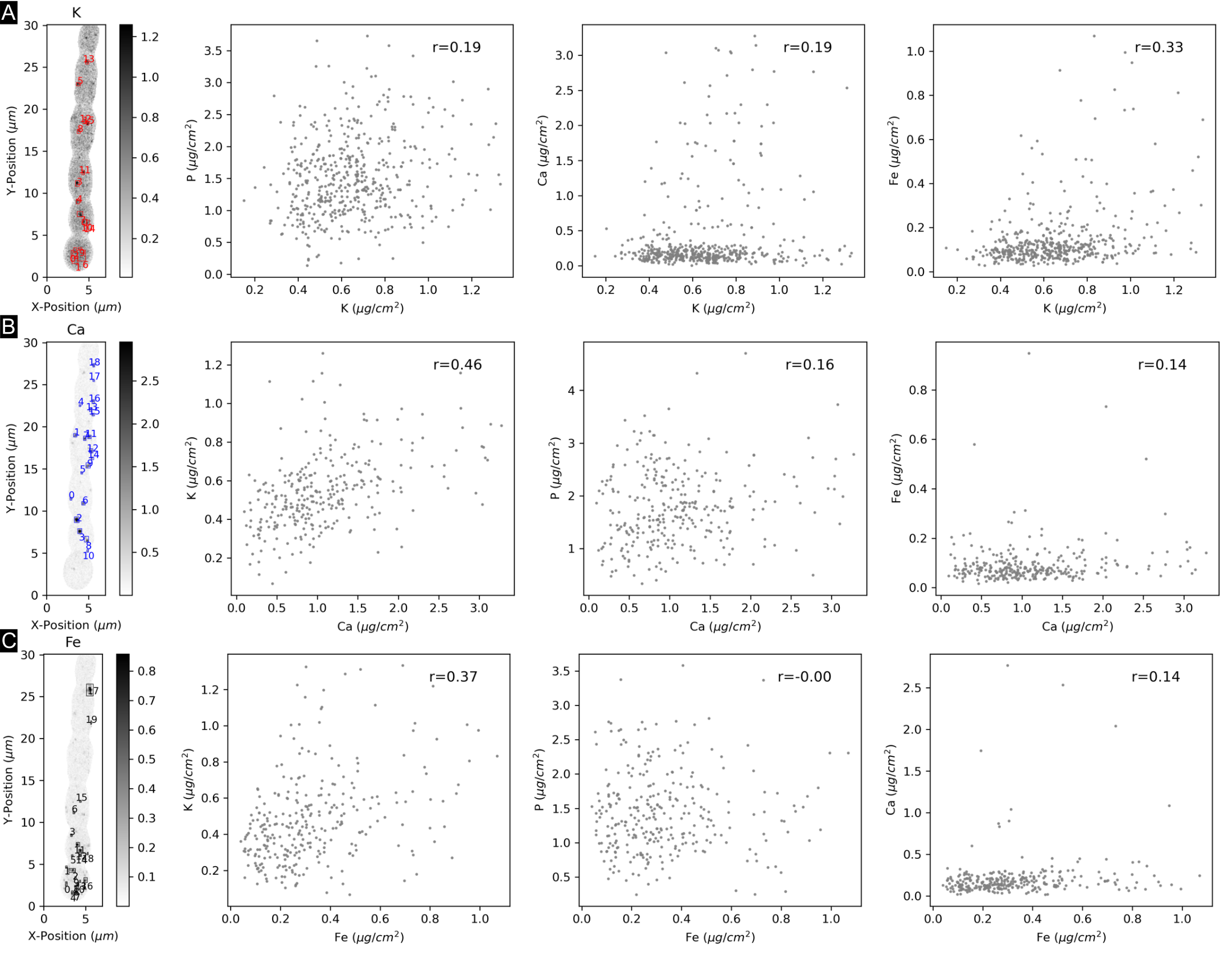

Supplement: mfae040_Supplemental_Files [file mfae040_supplemental_files.zip › Suppl_data_Figure_S7.tiff]

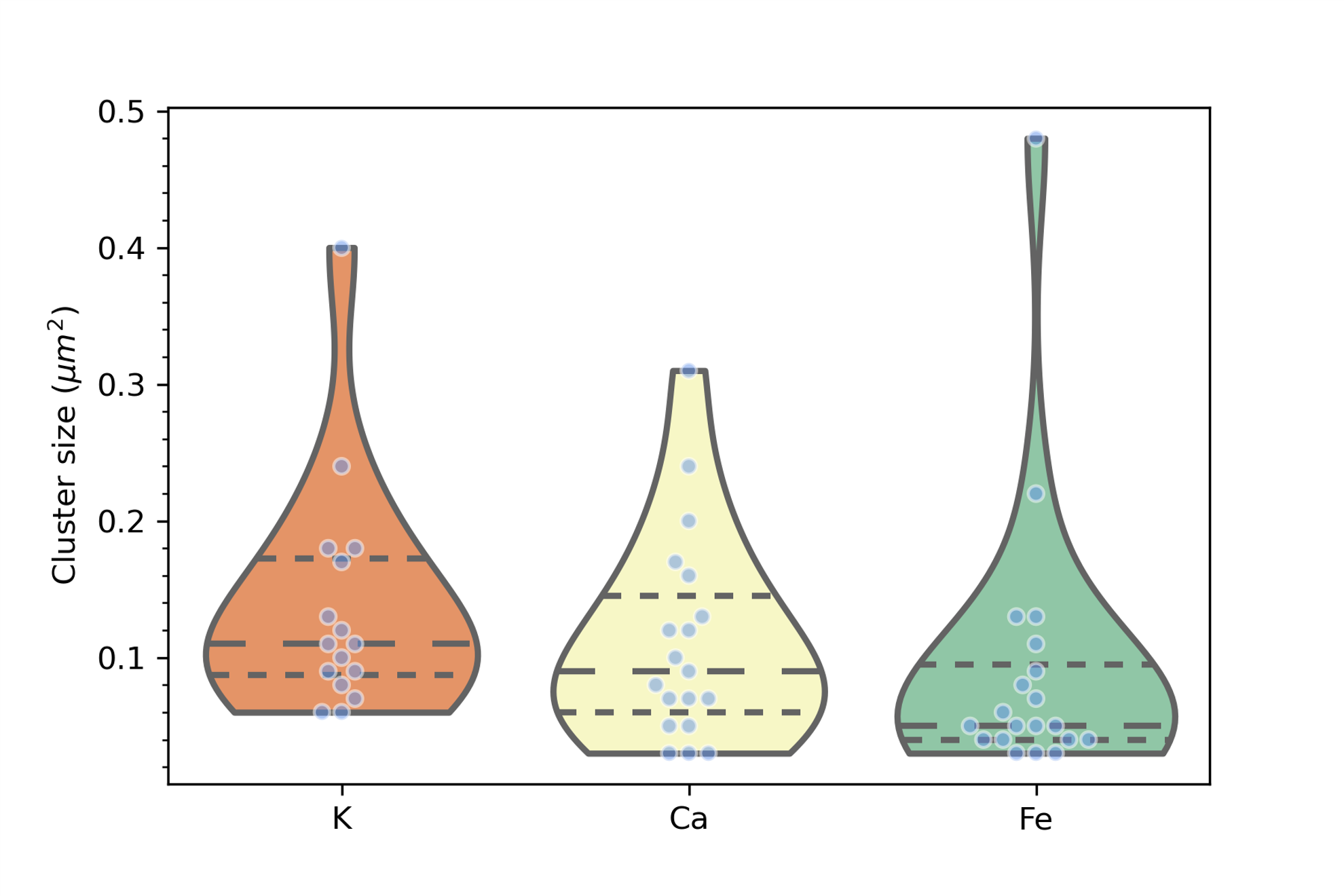

Supplement: mfae040_Supplemental_Files [file mfae040_supplemental_files.zip › Suppl_data_Figure_S8.tif]

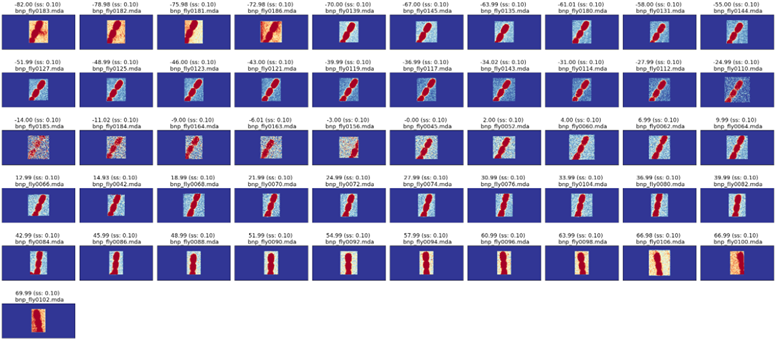

Supplement: mfae040_Supplemental_Files [file mfae040_supplemental_files.zip › Suppl_data_Figure_S9.tif]

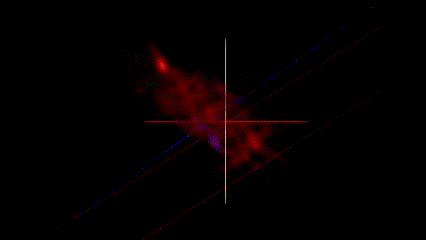

Supplement: mfae040_Supplemental_Files [file mfae040_supplemental_files.zip › Suppl_data_K-Ca_video.gif]
